# SSE forecasts based on market–sentiment dual anchoring

**DOI:** 10.1371/journal.pone.0339065

**Published:** 2025-12-26

**Authors:** Lei Yang, Bo Gan, Xueyan Niu, Qing Liu

**Affiliations:** 1 Department of Business Administration, Shandong Labor Vocational and Technical College, Jinan, China; 2 School of Economics and Management, Jingdezhen University, Jingdezhen City, Jiangxi Province, China; 3 College of Economics and Management, Huainan Normal University, Huainan, China; 4 Graduate School of Management of Technology, Pukyong National University, Busan, South Korea; Hanyang University, KOREA, REPUBLIC OF

## Abstract

Anchoring is widely considered one of the most robust and consistently observed effects in experimental psychology. This study employs the highest and lowest indices of the Shanghai Stock Exchange (SSE) alongside the highest and lowest bullish sentiments over a 52-week period as anchors, in conjunction with Fibonacci retracement levels, to develop a dual market–sentiment anchoring multivariate feature matrix. Based on this feature matrix, we propose a forecasting model called Market Sentiment Dual Anchoring CNN2D-ABiLSTM (MSD-CNN2D-ABiLSTM). This model employs CNN2D to extract spatial features from market and sentiment data, utilizes BiLSTM networks to process and integrate temporal features, and incorporates an attention mechanism to emphasize essential spatial and temporal information. Experimental results indicate that this model achieves a prediction accuracy exceeding 90% and an R^2^ value greater than 95% for lags of 1–2 trading days, enabling precise forecasting of the SSE index. Additionally, the model demonstrates effective forecasting performance for up to 10 trading days ahead, significantly outperforming traditional baseline models. Furthermore, structural sensitivity tests reveal that the extraction of local spatial features by CNN2D provides a predictive advantage over the short-term temporal features captured by CNN1D in complex market structures.

## 1. Introduction

Forecasting future stock market movements has historically been regarded as a significant and formidable task [1–5]. Forecasting the movements of the Shanghai Stock Exchange (SSE) Composite Index is notably challenging owing to its lax regulatory standards, significant volatility, and recurrent instances of manipulative trading [6].

The Shanghai Stock Market, founded on November 26, 1990, was China’s first and most prominent stock market, in which many of the nation’s cornerstone enterprises, major industries, and high-tech corporations are listed after rigorous evaluation. The SSE Composite Index can be viewed as a barometer of the Chinese stock market [6]. The ability to accurately and effectively predict the swings of the SSE Composite Index is valuable to financial investors, researchers, and China’s financial regulators [4].

Econometricians have utilized diverse methodologies to forecast stock market trends, including time-series analysis, conventional machine learning models, and deep learning models [7]. The initial features employed for stock price forecasting were restricted to fundamental stock data and a select number of price indicators [8,9]. Advancements in Internet technology have rendered text-mining approaches more complex, integrating textual data, such as investor sentiment and news, into datasets. Researchers have progressively included non-financial data, such as environmental and climate information, in stock price forecasts [4]. Advanced model architectures and comprehensive datasets have emerged as essential tools for researchers aiming to achieve precise stock price forecasts. Nevertheless, “anchoring” elements, which are essential for predicting market patterns, are frequently overlooked by stock market predictors.

Anchoring is considered one of the most robust and well-documented cognitive effects in experimental psychology [10]. In the financial domain, anchoring often manifests when investors overly rely on previous prices, historical data, or psychological expectations when making decisions regarding stock prices or market trends. Even when new information becomes available, individuals tend to unconsciously use this initial information as a reference point, adjusting their judgments around this “anchor.” Although anchoring may merely constitute a cognitive bias, it does provide valuable information for forecasting [11].

Financial literature has documented extensive evidence of implicit trading signals associated with anchoring. For instance, one study utilized investors’ tendency to anchor to the 52-week high to explain price momentum [12], whereas another demonstrated that investors, managers, and boards often use past peak prices as reference points when determining the value of target companies [13]. It has also been suggested that investors’ limited attention is one reason why they rely on simple anchoring information when making investment decisions [14]. In conclusion, existing research has documented that anchoring prices carry significant investment signals that can, to some extent, explain market trends. However, stock price forecasting has rarely incorporated anchoring, primarily applying it to investment decision-making.

Moreover, anchoring is often tied to a specific price point and is rarely associated with investor sentiment. However, in the Chinese stock market, retail investors account for 80–90% of the trading volume, with a significant proportion being inexperienced newcomers to stock investment [6]. Consequently, the Chinese stock market is characterized by greater uncertainty and volatility, implying that investor sentiment plays a significant role in predicting the trends of the SSE Composite Index.

To address these issues, we propose an MSD-CNN2D-ABiLSTM model structure for predicting the SSE Composite Index: (1) We create more detailed technical features based on fundamental price indicators, using Fibonacci retracement levels as market price anchors to better capture price movements and patterns. (2) We create an effective sentiment index using 4.4095 million investor texts and then design sentiment-anchoring technical features to investigate the impact of market sentiment on stock price movements. (3) We use a cutting-edge CNN2D-ABiLSTM network design, in which the CNN2D component detects local spatial aspects in market and sentiment data while a bidirectional long short-term memory (BiLSTM) network processes temporal features. This combination enables the model to comprehensively recognize complex market patterns.

This study offers three main contributions:

(1) We develop a more comprehensive feature matrix with dual anchoring in market and sentiment data. This matrix fully covers market price changes and mood influences, as well as market heterogeneity levels.
(2) We create a neural network model using the CNN2D-ABiLSTM architecture, which combines the ability of CNN2D to extract spatial features with the ability of BiLSTM to process temporal features. By incorporating an attention mechanism, the model enhances its focus on critical information, achieving accurate predictions for the next 10 trading days of the SSE Composite Index.
(3) Through structural sensitivity experiments, feature ablation studies, and sliding window sensitivity tests, we provide valuable guidance on network architecture, feature selection, and window choice, offering key references for future studies.

In conclusion, this study introduces a dual anchoring framework that integrates market and sentiment dimensions into SSE forecasting. By combining market- and sentiment-based anchoring within a unified methodological structure, the proposed approach attempts to capture both rational and emotional dynamics of market behavior. This framework seeks to improve the interpretability and robustness of forecasting models and provide potential insights into sentiment-driven market mechanisms.

The remainder of this paper is structured as follows: Section 2 examines the pertinent literature associated with this study. Section 3 delineates the dataset employed and elucidates the methodology used to create the sentiment index. Section 4 presents the features and model, providing an overview of the feature set and model architecture employed in this research. Section 5 presents the main experimental results. Section 6 explores the implications and significance of the findings. Finally, the conclusion summarizes the study.

## 2. Literature review

### 2.1. Methodology for forecasting stock prices

Research on stock price prediction is categorized into two primary approaches: fundamental and technical analyses [7,15]. Fundamental analysis attempts to forecast stock price movements by studying a company’s financial health, macroeconomic data, and industry development trends [16]. This technique focuses on determining a company’s intrinsic value, assuming that the market price of a stock will eventually reflect its genuine value [17]. Analysts must possess a deep understanding of financial statements, industry dynamics, and macroeconomic trends, as well as the ability to evaluate market trends and a company’s long-term growth prospects [18]. Fundamental analysis, in general, focuses on long-term patterns and is appropriate for medium- to long-term investors [7].

Technical analysis relies on historical price data to identify price trends and predict future market dynamics using graphical tools, mathematical methods, and econometric models [19]. This approach is predicated on three core assumptions: first, that market prices already reflect all available information, thus eliminating the need for additional fundamental analysis; second, that costs follow certain trends, implying that price movements in a given period will exhibit some directional bias; and third, that history tends to repeat itself, suggesting that past price behavior patterns may recur in the future [20]. The advantage of technical analysis lies in its ability to quickly identify short-term market opportunities, making it widely used in short-term trading and speculative activities. Technical analysis strategies are extensively applied in the field of stock price prediction, serving as a primary tool for many investors and researchers [7].

Researchers use various technical models for stock price prediction, which can be broadly categorized into time-series analysis, traditional machine learning models, and deep learning models [7]. Time-series analysis encompasses models such as autoregressive, moving average, autoregressive moving average, and autoregressive integrated moving average (ARIMA) models [21]. However, these models have limitations owing to their reliance on the assumptions of linearity and stationarity, which may not effectively capture the complex nonlinear relationships, volatility clustering, and non-stationary dynamics inherent in financial markets [22]. Similarly, traditional machine learning models, including regression analysis [23], support vector machines [24], random forests [25], and naive bayes [26], encounter challenges in dealing with the nonlinear and highly dynamic nature of financial markets. These models often struggle to adapt to rapidly changing market conditions and exhibit significant data dependency, limiting their applicability and predictive effectiveness.

To overcome the constraints of conventional models, numerous innovative machine learning methodologies, particularly neural network models, have been developed for forecasting financial time series. Neural networks possess robust nonlinear fitting capabilities, enabling them to identify concealed patterns in financial markets [27]. Deep learning methods are particularly adept at managing the non-stationarity and dynamic attributes of financial time series, yielding more precise and dependable models for stock price prediction [28]. The advent of recurrent neural networks (RNNs) and long short-term memory (LSTM) networks has markedly improved the comprehension and forecasting ability regarding intricate financial markets.

In recent years, BiLSTM, a novel variant of LSTM, has been extensively applied to both classification and regression tasks [1,29]. BiLSTM combines both forward and backward LSTM, enabling the model to not only leverage the ability of LSTM to capture long-term dependencies but also gather more comprehensive information by encoding the data in both directions [30]. Given that stock markets are inherently dynamic, nonlinear, volatile, noisy, and chaotic systems [31], the BiLSTM model is more adept than traditional LSTM models at handling nonlinear data, such as stock market trends. When BiLSTM integrates with attention mechanisms, it demonstrates a powerful ability to capture the nonlinear and complex characteristics of stock markets [29].

In addition, studies have shown that combining a CNN with BiLSTM not only facilitates the identification of spatial features in the market but also enhances the ability to discern both short- and long-term dependencies in time series using the BiLSTM model [1]. This integration enables the model to more comprehensively identify complex patterns in the market, significantly improving the accuracy and robustness of stock price predictions [8]. CNNs are particularly effective at capturing spatial features in the market and excel in handling high-dimensional data with complex interactions, such as price and market sentiment [1,32]. Therefore, this study combined a CNN, BiLSTM, and attention mechanisms to forecast the SSE Composite Index.

### 2.2. Market anchoring

It has been highlighted that investors’ limited attention partially accounts for their reliance on simple anchoring information when making investment decisions [14]. Numerous studies have confirmed the effectiveness of anchoring points as a tool for price prediction. Investors typically rely on past price anchors for investment decisions, a strategy that proves particularly effective during periods of market volatility [33].

Most stock price prediction research has focused on the impact of macroeconomic variables, technical indicators, or market sentiment on market trends. Despite the widespread use of anchoring prices in financial practice, their application in technical stock price forecasting has not received significant attention in academic research [33,34]. This is partly due to the simplicity of anchoring price features, which often consist of a single data point, such as the highest, lowest, or median price over a period, making it difficult to provide rich and robust data features [35].

Additionally, existing research shows that in addition to inherent market characteristics, market heterogeneity influences the relationship between technical features and stock price forecasting ability. These heterogeneous features include factors such as firm size [36], market volatility cycles [37], the proportion of retail investors [38], and price and return levels [39]. Therefore, this study seeks to link price anchoring with market heterogeneity by assessing the position of the current market price within Fibonacci levels over a given period while simultaneously evaluating market heterogeneity and price anchoring.

### 2.3. Sentiment anchoring

Historical research has demonstrated that investor sentiment plays a crucial role in stock price prediction, as emotions often influence investor decision-making, which, in turn, significantly impacts stock prices [37,40–42]. When sentiment is high, investors are more likely to buy stocks, driving prices upward; conversely, when sentiment is low, traders tend to sell, causing costs to decline [43]. Consequently, sentiment indicators are considered effective in enhancing the accuracy of stock price predictions. For example, it was found that investor sentiment is a key driver of short-term stock price fluctuations [44]. By analyzing sentiment indicators, researchers can better capture market volatility and improve the precision of future stock price forecasts [45].

Moreover, compared with institutional investors, retail investors are generally more susceptible to emotional fluctuations owing to their lack of systematic investment strategies [36]. Retail investors dominate the Chinese stock market, which is one of its unique characteristics. According to [46], an estimated 80–90% of market participants in the Chinese stock market are retail investors. This leads to greater market volatility, which is heavily driven by sentiment [44]. Therefore, incorporating sentiment indicators in predicting the SSE Composite Index is crucial [47].

Furthermore, previous studies have shown that market heterogeneity shapes the relationship between sentiment and stock price predictability, rather than being solely determined by sentiment. Previous evidence indicates that sentiment-driven predictability is most evident under extreme emotional conditions, implying that investor sentiment interacts dynamically with market structure and volatility [39]. However, existing studies tend to analyze market anchoring and sentiment effects separately, lacking an integrated framework that captures their joint influence on stock price dynamics. Therefore, anchoring the current emotional state of the market has been recognized as critical for understanding stock index movements [48].

Considering the above research gap, this study introduces the concept of dual anchoring, extending the anchoring mechanism from market prices to sentiment features and unifying the two within a single methodological structure. By developing a composite feature matrix for the SSE Composite Index that integrates both market and sentiment indicators, this framework captures the combined effects of rational market signals and emotional fluctuations. The proposed model, based on a CNN, BiLSTM, and attention mechanisms, leverages this market–sentiment dual anchoring to improve interpretability and predictive robustness in SSE forecasting.

## 3. Data

The primary data included in this study were categorized into two segments:

(1) Historical data of the SSE, which encompassed fundamental price indicators, such as daily opening prices, closing prices, and returns. These data were utilized to create the market feature matrix to forecast the SSE Composite Index.
(2) Investor commentary, which encompassed written financial remarks and their dissemination attributes, such as text, reading, and comment volumes. These data were utilized to construct the sentiment feature matrix for forecasting the SSE Composite Index.

### 3.1. Market data

We gathered market data for the SSE Composite Index for 1,214 trading days from January 2019 to December 2023. The dataset comprised daily statistics, such as opening prices, closing prices, highest prices, lowest prices, and returns. All historical market data were obtained from the official financial interface of Sina Finance (https://quotes.sina.cn/hs/company/quotes/view/sh000001), which provides publicly accessible and verifiable data for the SSE Composite Index. The data were downloaded and processed using Python for cleaning and standardization. Stock returns were calculated based on daily closing prices. Following [49], the daily return $R_{t}$ was defined as


$$R_{t}=\frac{P_{t}-P_{t-\text{1}}}{P_{t-\text{1}}}$$

where $P_{t}$ and $P_{t-\text{1}}$ represent the closing prices of the SSE Composite Index on days t and t−1, respectively. We utilized these data to establish the essential price and technical attributes for forecasting the SSE Composite Index. Table 1 presents the pertinent statistical descriptions.

**Table 1:** Statistical description of the SSE.

Variables	Opening price	Closing price	Highest price	Lowest price	Stock returns
mean	3,349.05	3,351.06	3,369.81	3,328.31	0.02
std	268.12	267.35	268.14	265.92	1.06
min	2,561.18	2,580.43	2,605.69	2,555.84	−7.72
25%	3,133.79	3,133.72	3,154.30	3,114.25	−0.52
50%	3,370.25	3,369.12	3,392.56	3,350.72	0.02
75%	3,557.89	3,558.98	3,580.76	3,531.02	0.60
max	3,900.81	3,894.03	3,911.92	3,870.38	5.71

*Note:* 25%, 50%, and 75% are quartile samples. RMB is the price unit. The variable “Stock returns” represents the daily simple return of the SSE Composite Index, computed using the formula above. The values highlighted in red within this column represent negative returns, indicating days of market decline.

### 3.2. Text data

In the “SSE Bar” (https://guba.eastmoney.com/) of the “Stock Bar,” numerous investors and organizations exchange their perspectives and analyses of the SSE on a daily basis. Each message typically includes the title, author, posting time, readership count, comment count, and textual content. Using Python-based web crawler scripts developed with the BeautifulSoup and urllib libraries, we collected publicly available investor posts from the “SSE Bar” on Eastmoney’s Stock Bar platform (https://guba.eastmoney.com/list,sh000001.html). The collection covered a five-year period (January 2019–December 2023). To comply with ethical standards and privacy protection, we retrieved only non-personally identifiable information, including timestamp, post content, reading count, and comment count. User names, IP addresses, and other personal metadata were strictly excluded.

The dataset comprised 4,409,500 distinct posts, which generated approximately 2,481 million views and 1,033,800 comments. On average, each post was viewed approximately 563 times, indicating a strong level of information diffusion and attention intensity within the SSE investor community. The reading and commenting volumes not only capture the extent of sentiment exposure and dissemination but also reflect interactive feedback among investors.

Data cleaning and preprocessing were conducted in Python, involving text normalization, timestamp alignment, and removal of duplicate or invalid entries to ensure data integrity and analytical consistency.

According to the framework proposed by [50], investor activity on social media can be viewed as a multilayered sentiment communication system: posting volume represents attention generation, reading volume reflects sentiment diffusion and exposure, and commenting volume indicates sentiment reinforcement through feedback. Together, these behavioral variables depict the dissemination and resonance of investor sentiment within the SSE online community, providing a dynamic behavioral foundation for sentiment-based market forecasting. Table 2 presents the statistical analysis of the text data.

**Table 2:** Statistical description of textual data.

Variables	Daily texts	Daily readings	Daily comments
mean	2,417	1,360,656	5,501
std	2,091	1,673,043	3,830
min	13	24,426	22
25%	573	585,589	3,064
50%	2,171	1,019,762	4,775
75%	3,323	1,637,714	6,846
max	19,204	44,924,350	43,562

***Note***: 25%, 50%, and 75% are quartile samples.

### 3.3. Bullish and agreement indices

After the investor commentary texts are acquired, they are used to construct an investor sentiment index, which generally involves two key steps: text sentiment classification and sentiment aggregation [51,52]. The first step involves assigning a sentiment orientation to each financial text, categorizing them as “positive,” “neutral,” or “negative.” The second step calculates the overall sentiment tendency of investors over a specific period by statistically counting the number of texts in each sentiment category. In this study, all 4,409,500 sentiment-classified text records—including original posts, readings, and comments from the SSE Bar—were incorporated into the computation of the investor sentiment (IS) and agreement (AG) indices.

**Step 1. Text sentiment classification**

Given the vast volume of investor social media posts (4,409,500), manual classification was impractical. Therefore, we adopted a supervised learning approach to assign sentiment labels—positive, neutral, or negative—to each post. The labeled dataset was manually constructed by the research team. Three team members independently annotated a randomly sampled subset of investor posts, and only the labels on which all three annotators agreed were considered valid. This procedure ensured labeling consistency and minimized subjective bias. In total, 60,000 posts were manually labeled, of which 50,000 were used for model training and 10,000 were reserved for independent accuracy validation. Labeled training data were used to teach the model how to detect relationships between textual content and sentiment categories [53].

Although the proportion of labeled data (approximately 1.36% of the total corpus) may appear relatively small, high-quality manual annotation in large-scale financial text corpora is inherently labor-intensive. Nonetheless, this labeling scale is consistent with benchmark studies in financial sentiment research, such as those conducted by [54,55], wherein a small yet carefully annotated dataset effectively supported robust model training and generalization. Hence, the sample size used in this study was considered sufficient and academically comparable for reliable sentiment modeling.

We developed a deep learning model that combines LSTM networks with self-attention mechanisms, both of which are widely used in sentiment classification tasks [29,56]. The model achieved an overall classification accuracy of 90.97% on the independent validation set, outperforming those of [57] at 89.14% and [54] at 88.1%. This result suggests the absence of systematic classification bias. The performance metrics are presented in Appendix A.

To enhance model reliability and address the potential for manipulation or misinformation in social media content, we implemented a structured text preprocessing pipeline prior to classification. Following [58,59], we removed extraneous characters, standardized usernames and URLs, and filtered out noisy expressions to improve semantic clarity. Rather than excluding potentially ambiguous or misleading texts, we allowed the model to classify them—typically into the neutral category—as suggested by [54]. This preserved the integrity of the dataset while enabling the model to handle information heterogeneity.

**Fig 1:**
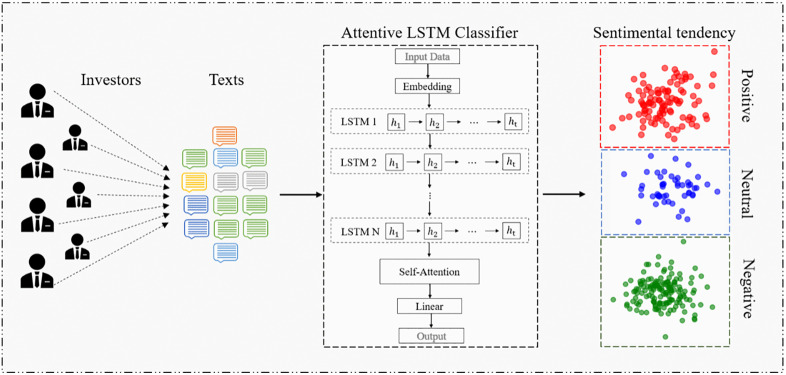
Flowchart of text sentiment classification. ***Note:*** outlines the pipeline from raw text preprocessing to supervised classification. To address concerns regarding manipulation and semantic ambiguity in user-generated content, we adopted a robust preprocessing strategy and used a neutral category to accommodate texts with unclear or noisy expressions. This enhances both classification robustness and data quality transparency.

Fig 1 illustrates the complete process and architecture of the proposed text sentiment classification framework.

**Step 2. Sentiment aggregation**

Following the advice of [52], we employed the approach suggested by [54] to develop the IS and AG indices. We specifically denoted the quantity of texts conveying positive sentiment during period t as $N_{t}^{+}$ and the quantity of texts conveying negative sentiment during period t as $N_{t}^{-}$. This enabled us to construct the IS index as follows:

${is}_{t}=ln\left[\frac{1+N_{t}^{+}}{1+N_{t}^{-}}\right]$.

This index, also referred to as the “bullish” index, reflects investor confidence during a specific period.

Investor opinions in the st68ock market often exhibit significant divergence. To quantify this divergence, [54] introduced the AG index, defined as

${ag}_{t}=1-\sqrt{1-{\left(\frac{N_{t}^{+}-N_{t}^{-}}{N_{t}^{+}+N_{t}^{-}}\right)}^{2}}$.

When all financial texts express bullish or bearish sentiment, AG equals 1, indicating complete agreement among investors. Conversely, when 50% of investors are bullish and 50% are bearish, investor disagreement reaches its maximum, yielding AG = 0. Table 3 presents a statistical description of the IS and AG indices constructed in this study.

**Table 3:** Statistical description of sentiment and agreement indices.

Variables	IS	AG
mean	−0.3524	0.0329
std	0.3902	0.0322
min	−1.3754	0.0000
25%	−0.6533	0.0063
50%	−0.3850	0.0225
75%	−0.0879	0.0522
max	0.9886	0.2013

***Note***: 25%, 50%, and 75% are quartile samples.

## 4. Feature and model development

### 4.1. Features description

This section delineates the procedure for creating a composite feature dimension that amalgamates essential market data with sentiment indicators, highlighting the dual anchoring properties of both market and sentiment elements.

#### 4.1.1. Market features.

A **Base price features**

Studies on technical models for forecasting stock price trends indicated that market prices comprehensively incorporate all accessible information, making historical price data vital for understanding investor behavior and market conditions [7,15]. It has been identified that the opening price, closing price, highest price, lowest price, and return are the essential characteristic parameters for forecasting the trend of the SSE [9,30]. Furthermore, to evaluate the extent of intraday price volatility, we computed the difference between the intraday high and low values.

The historical price series of the SSE Composite Index can be described as follows:


$$P=\left\{P_{t}|t=\text{1},\text{2},\text{3},\ldots ,T\right\}.$$

An observation point can be represented as


$$P_{t}=[p_{t}^{o},p_{t}^{h},p_{t}^{l},p_{t}^{c},p_{t}^{v},r_{t}]$$ (1)

where $p_{t}^{o},p_{t}^{h},p_{t}^{l},p_{t}^{c},p_{t}^{v},\;\text{and}\;r_{t}$ represent the opening price, highest price, lowest price, closing price, intraday volatility, and return characteristics at time t, respectively.

B **Bollinger bands features**

Fundamental price indicators offer only a basic understanding of market volatility patterns. Technical analysts believe that market trends are consistent and repeatable; thus, identifying technical indications may lead to more accurate forecasts of future prices [19]. Bollinger bands, developed by John Bollinger in 1980, are a technical analysis method that captures price patterns and generates trading signals by combining a middle band, an upper band, and a lower band [60]. Financial investors commonly employ Bollinger bands owing to their ability to clearly indicate market movements and identify trading opportunities [61].

Therefore, we constructed three sets of technical indicators: the Middle Band, Upper Band, and Lower Band. The Middle Band is typically represented by the simple moving average of the price series, calculated as follows:


$$MB=\frac{\text{1}}{n}\sum_{i=t-n+\text{1}}^{t}p_{i}^{c}$$

where $p_{i}^{c}$ is the closing price at time point i and n is the calculation window for the smoothing curve. The Upper Band and Lower Band are constructed based on the Middle Band and the standard deviation of prices:


UB=MB+(k×Std),

and


LB=MB−(k×Std).

where k is a constant (usually set to 2) and Std represents the standard deviation of closing prices over the statistical period.

**Fig 2:**
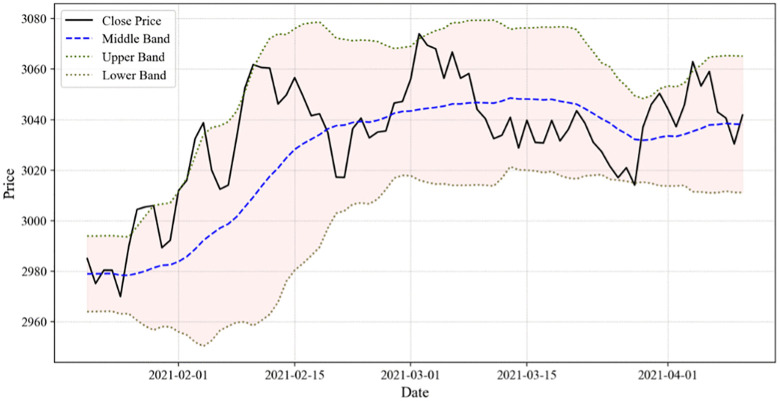
Bollinger band simulation curve. ***Note***: illustrates the structure of Bollinger bands, where the upper and lower bands define dynamic price boundaries relative to the moving average. This visualization reveals potential overbought and oversold zones, reflecting short-term market volatility and trend shifts.


Fig 2 shows that the Middle Band mitigates price volatility and indicates the medium-term market trend. The Upper Band represents a high-pressure area, indicating the likelihood of overbought conditions when prices are near or beyond this threshold. Conversely, the Lower Band signifies a low support zone, suggesting possible oversold situations when prices approach or fall below this threshold. We can delineate the technical feature set of the Bollinger bands as follows:

$$B_{t}=\left[{mb}_{t},ub_{t},lb_{t}\right]$$ (2)

where ${mb}_{t},ub_{t}$, and $lb_{t}$ represent the observed values of the Middle Band, Upper Band, and Lower Band at time point t, respectively. These technical indicators provide deeper insights for our market analysis.

C **Anchoring of market price levels**

The Fibonacci sequence is an infinite mathematical sequence [62], defined as follows:

**Initial definition:** The first two numbers in the sequence are 0 and 1, i.e., F(0)=0 and F(1)=1.

**Recursive relation:** For n>1, each number in the sequence is the sum of the two preceding numbers:


F(n)=F(n−1)+F(n−2).

As the numbers in the Fibonacci sequence increase, the ratio of two consecutive numbers approaches the golden ratio (φ≈0.618), the ratio of numbers spaced by one digit approaches the square of the golden ratio ($\varphi^{\text{2}}\approx \text{0}.\text{382}$), and the ratio of numbers spaced by two digits approaches the cube of the golden ratio ($\varphi^{\text{3}}\approx \text{0}.\text{236}$). The array [0,0.236,0.382,0.5,0.618,1] consists of the values φ, $\varphi^{\text{2}}$, $\varphi^{\text{3}}$, as well as the markers 0, 0.5, and 1. This array is often used to determine the trends in the stock market [63]. This technical analysis tool, known as Fibonacci retracement, helps traders identify probable support and resistance levels, enabling them to better capitalize on trading opportunities [64].

We achieve price anchoring and market-level assessment over the past 52 weeks by calculating the Fibonacci level of the current price relative to the price fluctuations during this period. We can determine the Fibonacci level (FL) of the current price within the 52-week high–low range using the following formula:


$$FL=\text{min}\left({\text{arg}\;\text{min}}_{f\in \left\{\text{0},\;\text{0}.\text{236},\;\text{0}.\text{382},\;\text{0}.\text{5},\;\text{0}.\text{618},\;\text{1}\right\}}|L-f|\right).$$

where L represents the ratio of the current price relative to the 52-week high–low range:


$$L=\frac{p_{t}^{c}-p_{min}^{c}}{p_{max}^{c}-p_{min}^{c}}$$

An L=0 indicates that the current price is the lowest within the 52-week period, whereas an L=1 indicates that the current price is the highest within the 52-week period.

The Fibonacci levels provide multiple anchors for market price levels, including the lowest and highest stock prices within the 52-week period, as well as the current market level. The Fibonacci retracement level feature ${PL}_{t}$ used to describe market price anchoring, can be expressed as


$${PL}_{t}=\left[{pl}_{t}\right].$$ (3)

#### 4.1.2. Sentiment features.

A **Sentiment communication features**

Financial text on social media exhibits two typical characteristics: user-generated content [65] and information communication [66]. User-generated content reflects investors’ attitudes toward investment, whereas content dissemination measures the spread of investor opinions within social networks. Research indicates that the volume of published text can directly reflect market sentiment and investor attention toward the market [51], whereas the volume of comments and replies serves as an effective indicator of the range of opinion dissemination.

Table 2 shows the distribution of daily investor texts. The significant differences in text, reading, and comment volumes on a given trading day reflect market sentiment dynamics, as well as the breadth and intensity of its communication. In this study, we employed investor text volume (${tv}_{t}$), reading amount (${rv}_{t}$), and reply comment volume (${cv}_{t}$) on trading days as the featured dimensions ($C_{t}$) to assess the communication of sentiment. These metrics were used in the SSE index prediction analysis. We define these communication feature dimensions as follows:


$$C_{t}=\left[{tv}_{t},{rv}_{t},{cv}_{t}\right].$$ (4)

B **Bullish index**

Retail investors account for 80–90% of the turnover in the Chinese stock market [6], which makes the IS index an important feature for predicting the SSE. We dedicate a significant amount of time to measuring the IS index ($is_{t}$) for each trading day. This index depicts the overall bullish sentiment of investors toward the SSE [43].

To further quantify the stage level of investor sentiment, we follow the anchoring logic described in Section 4.1.1 and calculate the daily sentiment level ($sl_{t}$) over a 52-week window using the Fibonacci retracement method. Specifically, we first identify the highest and lowest sentiment values during the past 52 weeks ($is_{max}$ and $is_{min}$) and compute the relative position of the current sentiment within this range.


$$L_{s}=\frac{is_{t}-is_{min}}{is_{max}-is_{min}}$$

We then compare $L_{s}$ with the standard Fibonacci ratio set {0, 0.236, 0.382, 0.5, 0.618, 1} and assign the closest ratio f as the sentiment level $sl_{t}$:


$$sl_{t}=\text{min}\left({\text{arg}\;\text{min}}_{f\in \left\{\text{0},\;\text{0}.\text{236},\;\text{0}.\text{382},\;\text{0}.\text{5},\;\text{0}.\text{618},\;\text{1}\right\}}\left|L_{s}-f\right|\right).$$

This index anchors the investor sentiment stage within the annual fluctuation range and identifies potential turning points or heterogeneity in market sentiment.

The IS index ($is_{t}$) and the sentiment level ($sl_{t}$) constitute the sentiment characterization dimension (${IS}_{t}$):


$${IS}_{t}=\left[is_{t},sl_{t}\right].$$ (5)

C **AG index**

In social media, investor sentiment often exhibits divergence, which constitutes a component of overall sentiment [67]. Based on this premise, we propose the Agreement Features Group ($A_{t}$) to assess the contribution of investor sentiment divergence to the prediction of the SSE Composite Index. We describe this new feature group as follows:


$$A_{t}=\left[{ag}_{t},al_{t}\right]$$ (6)

where ${ag}_{t}$ represents the consistency of investor sentiment at time t and $al_{t}$ denotes the Fibonacci-based level of investor sentiment consistency, calculated using the same method as $sl_{t}$ above. This alignment allows $al_{t}$ to anchor the temporal phase of agreement levels within the 52-week range, ensuring methodological consistency with sentiment and market anchoring.

#### 4.1.3. Problem formulation.

Table 4 shows the feature dimensions used in this study to forecast the SSE index. The first part of these feature dimensions originates directly from the stock market, which we call market features ($M_{t}$). Another part originates from investors’ opinions, which we call the sentiment group ($S_{t}$). This study predicts the SSE index based on market and sentiment dual feature anchoring.

**Table 4:** Summary description of feature variables.

No.	Group I	Group II	Feature	Description
1	Market ($M_{t}$)	Basic price ($P_{t}$)	$p_{t}^{o}$	The opening price on date t.
2			$p_{t}^{h}$	The highest price on date t.
3			$p_{t}^{l}$	The lowest price on date t.
4			$p_{t}^{c}$	The closing price on date t.
5			$p_{t}^{v}$	The closing volatility on date t
6			$r_{t}$	The return rate on date t.
7		Bollinger bands ($B_{t}$)	${mb}_{t}$	The middle band curve value on date t.
8			$ub_{t}$	The upper band curve value on date t.
9			$lb_{t}$	The lower band curve value on date t.
10		Price anchoring (${PL}_{t}$)	$pl_{t}$	The Fibonacci level for price on date t.
11	Sentiment ($S_{t}$)	Sentiment communication ($C_{t}$)	$tv_{t}$	The investor message volume on date t.
12			$rv_{t}$	The investor reading volume on date t.
13			$cv_{t}$	The investor comment volume on date t.
14		Sentiment and anchoring (${IS}_{t}$)	$is_{t}$	The investor sentiment index on date t.
15			$sl_{t}$	The Fibonacci level for investor sentiment on date t.
16		Agreement index ($A_{t}$)	${ag}_{t}$	The agreement index on date t.
17			$al_{t}$	The Fibonacci level for agreement index on date t.

*Note:* The sentiment communication variables ($tv_{t}$, $rv_{t}$, and $cv_{t}$) are derived from the daily counts of investor posts, readings, and comments collected from the SSE Bar. These three variables capture the generation, diffusion, and reinforcement of investor sentiment, respectively, in the online community, thereby quantifying the dynamic intensity of sentiment communication that contributes to the overall sentiment dimension ($S_{t}$).

Given a specific period, the feature dimension $F_{t}$ is defined as follows:


$$F_{t}=[M_{t},S_{t}]=[P_{t},B_{t},\;{{{PL}_{t},C}_{t},IS}_{t},A_{t}]$$ (7)

where $M_{t}$ represents the market feature set, comprising $P_{t}$ (price features), $B_{t}$ (Bollinger band features), and ${PL}_{t}$ (price levels); and $S_{t}$ denotes the sentiment feature set, encompassing $C_{t}$ (sentiment propagation features), ${IS}_{t}$ (investor sentiment features), and $A_{t}$ (AG index features). The feature collection used to predict the SSE can be represented as


$$F=\left\{F_{t}|t=\text{1},\text{2},\text{3},\cdots ,T\right\}.$$

We consider a time window of length w, spanning from time t−w+1 to time t, which includes the feature vectors from the past w days, expressed as


$$X_{w}=\left[F_{t-w+\text{1}},\cdots ,F_{t-\text{2}},F_{t-\text{1}},F_{t}\right].$$

Our objective is to leverage this feature vector $X_{w}$ to predict the closing prices of the SSE index for the subsequent lag days, denoted as


$$Y_{lag}=\left[p_{t+\text{1}}^{c},p_{t+\text{2}}^{c},\cdots ,p_{t+lag}^{c}\right].$$

### 4.2. Model

#### 4.2.1. Overall structure.

**Fig 3:**
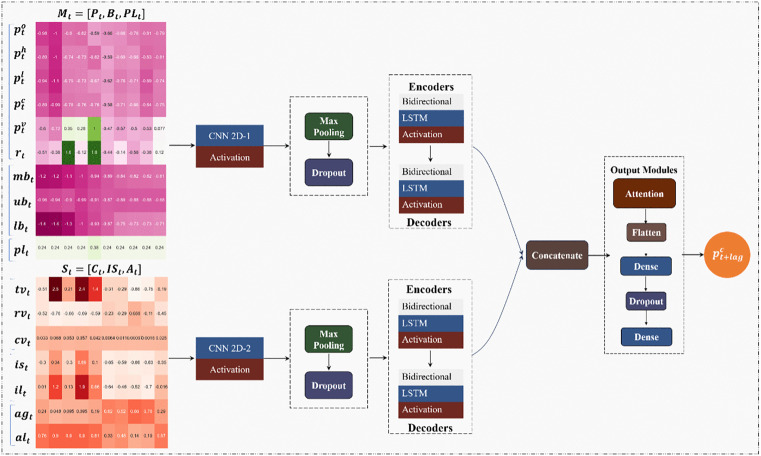
Overall structure of MSD-CNN2D-ABiLSTM. ***Note:*** The architecture of the MSD-CNN2D-ABiLSTM model, which integrates two CNN2D blocks (64 filters, 3 × 3 kernel, ReLU activation, 20% dropout, max pooling), BiLSTM encoders and decoders (50 units each), and an attention mechanism to capture temporal dependencies and assign dynamic weights to the features. The final output is generated through two dense layers (50 and 1 units) using ReLU and linear activations. The model is trained using the Adam optimizer (learning rate = 0.001) and MSE loss, balancing accuracy, interpretability, and computational efficiency.

Fig 3 illustrates the overall structure of the proposed model. In this section, we detail the logical framework of our model.

We first employ two CNN2D networks to separately load market indicator data (CNN2D_1) and sentiment indicator data (CNN2D_2) to extract local holistic spatial features. CNN2D effectively reduces the feature dimensions and compresses the information through convolution and pooling operations, which helps reduce the computational complexity and eliminate noise.

The key reason for utilizing CNN2D is its robust capability to handle graphical data and spatial visual information. The various market indicators, such as prices, Bollinger bands, and sentiment, are not isolated entities; rather, they are interconnected, collectively influencing the current market and sentiment states. Using CNN2D, we can conceptualize these indicators as a unified “image,” capturing the complex dependencies among features in a spatial context. This approach lays a solid foundation for subsequent model analyses.

After CNN2D extracts the features, a BiLSTM network encodes and decodes them, capturing the temporal dependencies and long-term patterns of market features. Next, we combine the outputs of the $M_{t}$ and $S_{t}$ feature sets and apply an attention mechanism to score and weight each feature. This process enables the model to focus more accurately on the features that are most critical for prediction.

After the weighted scoring, the output features undergo further processing through two dense layers to enable deep learning on the integrated features. To prevent overfitting, we incorporate Dropout layers, enhancing the model’s generalization ability. Finally, the model outputs the predicted value of the SSE Index after applying a linear activation function.

To enhance transparency and reproducibility, we included all major hyperparameter settings in the caption of Fig 3. These parameters—including filter sizes, dropout rates, and LSTM units—were tuned based on preliminary experiments and previous studies to ensure robust learning and generalization.

The dataset used in this study was constructed strictly in chronological order to ensure that each input window contained only information from the preceding time steps. Samples were generated using a sliding-window approach, where the input features were drawn from past periods and the prediction target corresponded to a future lag. For data partitioning, a non-shuffled, time-based holdout method with an 80:20 ratio was adopted, ensuring that the training set contained only earlier time segments, whereas the testing set consisted exclusively of later, unseen data. No random shuffling was performed, thereby preventing any form of data leakage or look-ahead bias. To confirm robustness, the same chronological partitioning strategy was applied consistently across all experiments, including ablation and sensitivity analyses, and was further validated through multiple forecast horizons (lags 1–20), effectively serving as a rolling evaluation framework.

#### 4.2.2. Module description.

A **Feature matrix**

The shape of the market feature matrix $M_{t}$ is given by $M_{t}\in R^{w\times \text{10}}$, where 10 represents the number of features and w denotes the feature window size. The shape of the sentiment feature matrix $S_{t}$ is defined as $S_{t}\in R^{w\times \text{7}}$, where 7 is the number of features and w is the feature window size. The overall feature matrix $F_{t}$ can be described as


$$F_{t}=[M_{t},S_{t}]$$

Therefore, the shape of $F_{t}$ is determined by $F_{t}\in R^{w\times (\text{10}+\text{7})}=R^{w\times \text{17}}$.

B **CNN2D module**

First, we perform 2D convolution operations separately on the market features $M_{t}$ and the sentiment features $S_{t}$ to extract their local spatial features in a graphical representation:


$$\begin{array}{c} M_{t}^{conv}=\sigma (W^{\left(M\right)}*M_{t}+b^{(M)})\\ S_{t}^{conv}=\sigma (W^{\left(S\right)}*S_{t}+b^{(S)}) \end{array}$$ (8)

where

• *W*^*(M)*^ and $W^{\left(S\right)}$ are the weight matrices of the convolution kernels for $M_{t}$ and $S_{t}$, respectively.
• *b*^*(M)*^ and$\;b^{\left(S\right)}$ are the bias terms.
• *σ* is a nonlinear activation function, for which this study adopts the rectified linear unit (ReLU) function.
C **Pooling and dropout operations**

After the convolution operations, the market features $M_{t}^{conv}$ and sentiment features $S_{t}^{conv}$ are passed through pooling layers for subsampling. This operation aims to reduce the spatial dimensions of the feature maps, thereby lowering the computational complexity and mitigating the risk of overfitting. We employ the Max Pooling operation with a pooling window size of p×p:


$$\begin{array}{c} M_{t}^{pool}=𝐦𝐚𝐱\left(M_{t}^{conv}\left(i,j\right)\right|i,j\in p\times p)\\ S_{t}^{pool}=𝐦𝐚𝐱\left(S_{t}^{conv}\left(i,j\right)|i,j\in p\times p\right) \end{array}.$$ (9)

The pooled market features $M_{t}^{pool}$ and sentiment features $S_{t}^{pool}$ are then passed to Dropout layers for regularization to prevent model overfitting. In the Dropout layer, each input neuron z is retained with a probability p and discarded with a probability 1−p:


$$\begin{array}{c} M_{t}^{drop}=M_{t}^{pool}⨀r^{(M,p)}\\ S_{t}^{drop}=S_{t}^{pool}⨀r^{\left(S,p\right)} \end{array}$$ (10)

where $r^{\left(M,p\right)}\sim Bernoulli(p)$ and ${\;r}^{\left(S,p\right)}\;\sim Bernoulli(p)$ represent independently sampled binary random variables that control the retention or dropping of neurons. The symbol ⨀ denotes element-wise multiplication.

D **BiLSTM encoding and decoding**

After pooling and Dropout operations, the market feature $M_{t}^{drop}$ and the sentiment feature $S_{t}^{drop}$ are passed to the BiLSTM network for encoding and decoding the temporal features, respectively. The BiLSTM captures the feature sequence by simultaneously performing both forward and backward pass computations. The sequence has – temporal dependencies. Managing time-series patterns specific to market and sentiment data is crucial because a combination of past and future events may influence market fluctuations and sentiment changes.

For the market features $M_{t}^{drop}$, the forward and backward processes during the encoding phase can be represented as follows:


$$\begin{array}{c} {\overset{⇀}{h}}_{t}^{(M,enc)}=\sigma (W_{f}^{\left(M\right)}\cdot M_{t}^{drop}+U_{f}^{\left(M\right)}\cdot \;{\overset{⇀}{h}}_{t-1}^{\left(M,enc\right)}+b_{f}^{\left(M\right)}\\ {\overset{↼}{h}}_{t}^{(M,enc)}=\sigma (W_{b}^{\left(M\right)}\cdot M_{t}^{drop}+U_{b}^{\left(M\right)}\cdot \;{\overset{↼}{h}}_{t+1}^{\left(M,enc\right)}+b_{b}^{\left(M\right)} \end{array}.$$ (11)

where

• ${\overset{⇀}{h}}_{t}^{\left(M,enc\right)}$ and ${\overset{↼}{h}}_{t}^{\left(M,enc\right)}$ are the hidden states of the forward and backward LSTM units, respectively.
• $W_{f}^{\left(M\right)}$ and $W_{b}^{\left(M\right)}\;$are the input weight matrices for the forward and backward LSTMs, respectively.
• $U_{f}^{\left(M\right)}$ and $U_{b}^{\left(M\right)}$ are the recurrent weight matrices.
• $b_{f}^{\left(M\right)}$ and $b_{b}^{\left(M\right)}$ are the bias terms.
• σ is the tanh activation function.

The forward and backward hidden states ${\overset{⇀}{h}}_{t}^{\left(M,enc\right)}$ and ${\overset{↼}{h}}_{t}^{\left(M,enc\right)}$ are concatenated to form the encoding output of the BiLSTM layer:

$M_{t}^{Bi-enc}=[{\overset{⇀}{h}}_{t}^{\left(M,enc\right)},{\overset{↼}{h}}_{t}^{\left(M,enc\right)}]$.

Subsequently, the encoded output $M_{t}^{Bi-enc}$ is input into another BiLSTM layer for decoding, extracting deeper temporal features. The forward and backward processes during the decoding phase are represented as follows:


$$\begin{array}{c} {\overset{⇀}{h}}_{t}^{(M,dec)}=\sigma (W_{f}^{\left(M,dec\right)}\cdot M_{t}^{Bi-enc}+U_{f}^{\left(M,dec\right)}\cdot \;{\overset{⇀}{h}}_{t-1}^{\left(M,dec\right)}+b_{f}^{\left(M,dec\right)}\\ {\overset{↼}{h}}_{t}^{(M,dec)}=\sigma (W_{b}^{\left(M,dec\right)}\cdot M_{t}^{Bi-enc}+U_{b}^{\left(M,dec\right)}\cdot \;{\overset{↼}{h}}_{t+1}^{\left(M,dec\right)}+b_{b}^{\left(M,dec\right)} \end{array}$$ (12)

The decoding process similarly merges the forward and backward hidden states ${\overset{⇀}{h}}_{t}^{\left(M,dec\right)}$ and ${\overset{↼}{h}}_{t}^{\left(M,dec\right)}$ to form the decoded result for market features:


$$M_{t}^{Bi-dec}=[{\overset{⇀}{h}}_{t}^{\left(M,dec\right)},{\overset{↼}{h}}_{t}^{(M,dec)}].$$ (13)

Similarly, we can obtain the decoded result for the sentiment features $S_{t}^{drop}$ as follows:


$$S_{t}^{Bi-dec}=[{\overset{⇀}{h}}_{t}^{\left(S,dec\right)},{\overset{↼}{h}}_{t}^{(S,dec)}].$$ (14)

E **Attention mechanism**

After the BiLSTM encoding and decoding process, the market features $M_{t}^{Bi-dec}$ and sentiment features $S_{t}^{Bi-dec}$ are merged into a single comprehensive feature vector:


$$F_{t}^{merged}=[M_{t}^{Bi-dec},S_{t}^{Bi-dec}].$$ (15)

where $F_{t}^{merged}\in R^{\text{d}}$ represents the combined temporal feature vector that integrates both market and sentiment features. This merged feature vector $F_{t}^{merged}$ is then passed into an attention mechanism layer.

**Attention score.** The importance of each feature, or attention score, is calculated using a linear transformation followed by a nonlinear activation function:


$$e_{t}=f\left(F_{t}^{merged},h\right)=𝐭𝐚𝐧𝐡(W_{attn}\cdot F_{t}^{merged}+b_{attn})$$ (16)

where

• $e_{t}\in R^{d}$ represents the unnormalized attention score.
• $W_{attn}\in R^{d\times d}$ is the learnable weight matrix.
• $b_{attn}\in R^{d}$ is the bias vector.

**Attention weights.** We apply the SoftMax function to normalize the attention score $e_{t}$, yielding the attention weights $\alpha_{t}$:


$$\alpha_{t}=\frac{\text{exp}(e_{t})}{\sum_{t=\text{1}}^{T}\text{exp}(e_{t})}$$

where $\alpha_{t}\in R^{d}$ is the normalized attention weight, satisfying $\sum_{t=\text{1}}^{T}\alpha_{t}=\text{1}$, which represents the importance of each feature at time step t for the final prediction.

**Attention-weighted feature representation.** The attention weights $\alpha_{t}$ are then applied to the merged feature vector $F_{t}^{merged}$, generating the final attention-weighted feature representation:


$$F_{t}^{attn}=\sum {\mathrm{\backslash nolimits}}_{t=1}^{T}\alpha_{t}\cdot F_{t}^{merged}$$ (17)

where $F_{t}^{attn}\in R^{d}$ is the feature vector weighted by the attention mechanism, which encapsulates critical information from both market and sentiment features, considering the temporal importance of each feature.

F **Prediction module**

In the prediction module, the feature vector $F_{t}^{attn}$, which has been processed by the attention mechanism, undergoes a series of transformations, including flattening, passage through two fully connected layers, dropout, and finally passage through the output layer to generate the final prediction.

**Flatten.** The feature vector $F_{t}^{attn}$, produced by the attention mechanism, is transformed into a one-dimensional vector via flattening as follows:


$$f_{t}^{flatten}={vec(F}_{t}^{attn})$$ (18)

where $f_{t}^{flatten}\in R^{d}$ is the flattened feature vector, which provides structured input data for subsequent fully connected layers.

**Dense layer 1.** The flattened vector $f_{t}^{flatten}$is passed into the first fully connected layer:


$$h_{1}=\sigma (W_{1}\cdot f_{t}^{flatten}+b_{1})$$ (19)

where $W_{\text{1}}$ is the weight matrix of the first fully connected layer, $b_{\text{1}}$ is the bias vector, and σ(·) is the activation function. In this study, we used the ReLU activation function.

**Dropout.** The output of the first fully connected layer $h_{\text{1}}$ undergoes dropout to further reduce the risk of overfitting:


$$h_{1}^{drop}=h_{1}\;⨀\;r^{(h_{1},p)}$$ (20)

where p is the dropout probability and $r^{\left(h_{\text{1}},p\right)}$ represents the randomly sampled binary mask.

**Dense layer 2.** The dropout-processed vector $h_{\text{1}}^{drop}$ is passed into the second fully connected layer:


$$h_{2}=\sigma (W_{2}\cdot h_{1}^{drop}+b_{2})$$ (21)

where $W_{\text{2}}$ is the weight matrix of the second fully connected layer and $b_{\text{2}}$ is the bias vector.

**Output layer.** The output of the second fully connected layer h2h_2h2 is passed through a linear activation function to generate the final prediction $\hat{y}$:


$$\hat{y}=W_{out}\cdot h_{2}+b_{out}$$ (22)

where $W_{out}$ is the weight matrix and $b_{out}$ is the bias vector of the output layer.

This model integrates the optimal features of convolutional, BiLSTM, attention, and fully connected layers. It effectively combines market and sentiment data to facilitate short-term forecasting of the SSE index.

## 5. Experiments

### 5.1. Evaluation metrics

We utilize the root mean squared error (RMSE), mean absolute error (MAE), coefficient of determination (R^2^), and direction accuracy (DA) to thoroughly assess the prediction efficacy of the model for the SSE Composite Index. These metrics evaluate the divergence between the actual value ($y_{i}$) and projected value (${\hat{y}}_{i}$) from various perspectives:

The RMSE measures the square root of the average squared error between the predicted and actual values, making it sensitive to large deviations or outliers in the predictions. This metric is particularly useful for evaluating the model’s ability to handle extreme values. The formula is as follows:


$$RMSE=\sqrt{\frac{\text{1}}{n}\sum {\mathrm{\backslash nolimits}}_{i=\text{1}}^{n}{\left(y_{i}-{\hat{y}}_{i}\right)}^{\text{2}}}$$

The MAE quantifies the average absolute error between the actual and predicted values. Compared with the RMSE, the MAE is less sensitive to outliers, making it suitable for measuring the overall error level in balanced datasets. The formula is as follows:


$$MAE=\frac{\text{1}}{n}\sum {\mathrm{\backslash nolimits}}_{i=\text{1}}^{n}\left|y_{i}-{\hat{y}}_{i}\right|$$

R^2^ reflects the goodness of fit of the model, indicating how well the predicted values align with the actual data. Its value typically ranges from 0 to 1, with values closer to 1 suggesting stronger explanatory power of the model. The formula is as follows:


$$R^{\text{2}}=\text{1}-\frac{\sum_{i=\text{1}}^{n}{\left(y_{i}-{\hat{y}}_{i}\right)}^{\text{2}}}{\sum_{i=\text{1}}^{n}{\left(y_{i}-\overset{―}{y}\right)}^{\text{2}}}$$

where $\overset{―}{y}$ is the mean of the actual values.

The DA measures the accuracy of the model in predicting the direction of price movements (upward or downward). This is particularly important in financial markets, where investors prioritize trend predictions over exact price estimations. The formula is as follows:


$$DA=\frac{\text{1}}{n}\sum_{i=\text{1}}^{n}I((y_{i+\text{1}}-y_{i})\cdot ({\hat{y}}_{i+\text{1}}-{\hat{y}}_{i})>\text{0})$$

where I is an indicator function that assumes a value of 1 when the condition is satisfied and 0 otherwise.

Together, these metrics provide a comprehensive evaluation of the model’s performance, encompassing the error magnitude, goodness of fit, and accuracy of trend predictions.

### 5.2. Comparison with baseline models

The performance of the proposed MSD-CNN2D-ABiLSTM model in predicting the trend of the SSE Composite Index was first compared with those of traditional baseline models. The baseline models include a wide range of classical machine learning and deep learning approaches commonly used in time-series forecasting and financial data analysis. These traditional models include statistical methods such as ARIMA; distance- and tree-based machine learning models such as KNN, decision trees, and random forests; and boosting techniques such as XGBoost. Deep learning models include RNNs, LSTM, and gated recurrent units (GRUs), along with more advanced models that integrate convolutional layers and attention mechanisms, such as Attention LSTM and spatio-temporal graph convolutional networks.

In forecasting the SSE index, we ensured robustness by fine-tuning the parameters of classical machine learning and time-series models through grid search. The feedforward neural network, which uses a three-layer architecture with ReLU activation to capture the nonlinear features of the data, was trained using the Adam optimizer in conjunction with the mean squared error loss function. Other deep learning models employ an encoder–decoder structure, incorporating Dropout operations to prevent overfitting, and are configured with relevant settings to preserve the temporal information inherent in time-series data.

Table 5 shows the baseline model’s prediction performance for the SSE after one day. The error metrics, specifically the RMSE and MAE, exhibit comparable performance across SVR, linear regression, decision tree, KNN, and random forest, indicating their effective data fitting capabilities. However, the accuracy of these models in predicting trend direction is relatively low, with most models having an accuracy range between 50% and 65%. The R² values for ARIMA and SVR are 0.8625 and 0.8604, respectively, indicating some degree of stability in accounting for market volatility; nevertheless, these models remain limited in their capacity for trend prediction.

**Table 5:** Comparison of the 1-day-ahead SSE forecasting performance of baseline models.

No.	Model	RMSE	MAE	R^2^	DA
1	ARIMA (Autoregressive Integrated Moving Average)	0.0617	0.0500	0.8625	0.5817
2	SVR (Support Vector Regression)	0.0535	0.0415	0.8604	0.6026
3	LR (Linear Regression)	0.0901	0.0739	0.7319	0.5183
4	KNN (k-Nearest Neighbors)	0.1303	0.1038	0.7645	0.5131
5	DT (Decision Tree)	0.1059	0.0824	0.7444	0.6183
6	RF (Random Forest)	0.0585	0.0443	0.8526	0.6183
7	XGBoost (eXtreme Gradient Boosting)	0.0588	0.0452	0.8520	0.5921
8	BR (Bayesian Regression)	0.0593	0.0455	0.8512	0.5764
9	VAE (Variational Autoencoder)	0.3724	0.3065	0.1000	0.5283
10	TCN (Temporal Convolutional Network)	0.1092	0.0880	0.8344	0.5869
11	ST-GCN (Spatio-Temporal Graph Convolutional Network)	0.2611	0.2087	0.1494	0.6806
12	FNN (Feedforward Neural Network)	0.0894	0.0664	0.8892	0.6497
13	CNN1D (Convolutional Neural Network 1D)	0.0459	0.0358	0.9005	0.7963
14	CNN2D (Convolutional Neural Network 2D)	0.0508	0.0410	0.9139	0.7539
15	RNN (Recurrent Neural Network)	0.0699	0.0556	0.9016	0.7639
16	LSTM (Long Short-Term Memory)	0.0397	0.0466	0.9186	0.8063
17	ALSTM (Attention Long Short-Term Memory)	0.0393	0.0439	0.9236	0.8548
18	GRU (Gated Recurrent Unit)	0.0616	0.0477	0.9173	0.5869
19	BiLSTM (Bidirectional Long Short-Term Memory)	0.0419	0.0489	0.9251	0.8288
20	MSD-CNN2D-ABiLSTM	0.0372	0.0312	0.9705	0.9153

***Note****:* All these experiments were performed with the feature window length set to 10.

In comparison, the deep learning models performed significantly better. Specifically, models such as CNN1D, CNN2D, LSTM, GRU, and BiLSTM outperformed the traditional models in both error metrics and trend direction prediction accuracy. Notably, LSTM and BiLSTM achieved R² values exceeding 0.9, with trend prediction accuracies above 80%, indicating that deep learning models can more effectively capture complex patterns in time-series data, resulting in stronger predictive capabilities.

**Fig 4:**
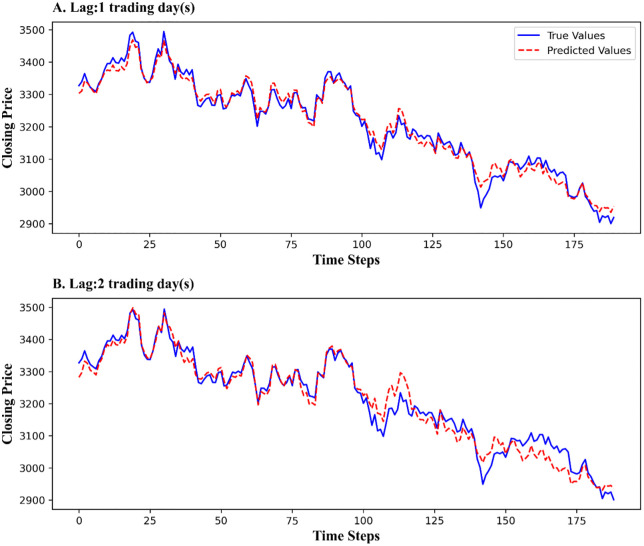
Forecasting curve of the MSD-CNN2D-ABiLSTM model for a 1- to 2-day lag SSE. ***Note***: Compares the predicted and actual values of the SSE Composite Index for 1- and 2-day forecast horizons. The close alignment between the two curves indicates that the proposed model effectively captures short-term market fluctuations and directional trends.

Our proposed MSD-CNN2D-ABiLSTM model excelled in all evaluation metrics, surpassing the baseline models. The MSD-CNN2D-ABiLSTM predicted the 1-day-ahead trend of the SSE with an R^2^ value of 0.9705 and a trend direction prediction accuracy of 91.53%, demonstrating exceptional forecasting performance. Fig 4 compares the predicted and actual values of the SSE Composite Index for a 1–2-day lag, highlighting the model’s superior short-term predictive capability.

### 5.3. Predicted performance over 20 days

Table 6 shows the predictive performance of the MSD-CNN2D-ABiLSTM model concerning closing prices ($p_{t+lag}^{c}\;|\;lag\in [\text{1},\text{20}]$), with lags ranging from 1 to 20 trading days. Because each week includes five trading days, this analysis effectively evaluates the model’s ability to predict closing prices over a period of approximately one month.

**Table 6:** Predictive performance of MSD-CNN2D-ABiLSTM for lags of 1–20 trading days.

Lag	RMSE	MAE	$R^{\text{2}}$	DA
1	0.0372	0.0312	0.9705	0.9153
2	0.0560	0.0433	0.9550	0.9043
3	0.0608	0.0478	0.9459	0.8289
4	0.0674	0.0503	0.9322	0.8280
5	0.1392	0.1074	0.7062	0.8207
6	0.0864	0.0660	0.8845	0.6885
7	0.1037	0.0718	0.8297	0.7912
8	0.1022	0.0800	0.8316	0.7790
9	0.1471	0.1141	0.6441	0.7667
10	0.1012	0.0775	0.8288	0.7247
11	0.1194	0.0954	0.7580	0.7006
12	0.1275	0.0993	0.7202	0.6307
13	0.1227	0.1025	0.7385	0.6686
14	0.2074	0.1480	0.2454	0.7069
15	0.2054	0.1696	0.2482	0.6570
16	0.2174	0.1711	0.1429	0.6199
17	0.2856	0.2458	−0.5042	0.5471
18	0.2431	0.2226	−0.1084	0.6450
19	0.2713	0.2094	−0.3901	0.5298
20	0.3292	0.2420	−1.0557	0.5783

***Note****:* Because each week includes five trading days, the 20 trading days represent the prediction for the entire four-week period. The length of the feature window was set to 10.

(1) **Short-term predictive performance (Lag = 1–5)**

As shown in Table 6, the model demonstrates excellent performance in short-term forecasts for the SSE. At a lag of 1, the RMSE is 0.0372, the MAE is 0.0312, and the R^2^ reaches 0.9705, with a DA of 91.53%. These results indicate that the MSD-CNN2D-ABiLSTM model effectively captures short-term market fluctuations and is well-suited for predicting short-term trends. As the lag increases from 2 to 4, the error remains within an acceptable range, although the RMSE and MAE slightly increase to 0.0674 and 0.0503, respectively. R^2^ remains above 0.93, and the DA remains between 82% and 90%, demonstrating the model’s robustness in short-term forecasting.

However, at a lag of 5, the predictive performance declines. Although the DA remains at 82.07%, the error increases, potentially reflecting biases in the model when weekend or holiday effects are considered, given that five trading days span a full trading week.

(2) **Mid-term predictive performance (Lag = 6–10)**

As the lag increases further, the prediction error continues to increase. At a lag of 6, the RMSE rises to 0.0864, and the R^2^ drops to 0.8845. Despite the growing error, the R^2^ at a lag of 10 remains 0.8288, and the DA remains above 70%, suggesting that the model retains a certain level of predictive ability for mid-term forecasts.

(3) **Long-term predictive performance (Lag = 11–20)**

Starting from a lag of 11, the model’s predictive performance deteriorates significantly. When the lag surpasses 15, the R^2^ approaches zero or even becomes negative, indicating the model’s limited capacity to explain market fluctuations during this period. At a lag of 17, the R^2^ is −0.5042, revealing potential systematic biases in the model’s long-term predictions. Additionally, the DA drops to the 50%–60% range, approaching the level of random predictions.

**Fig 5:**
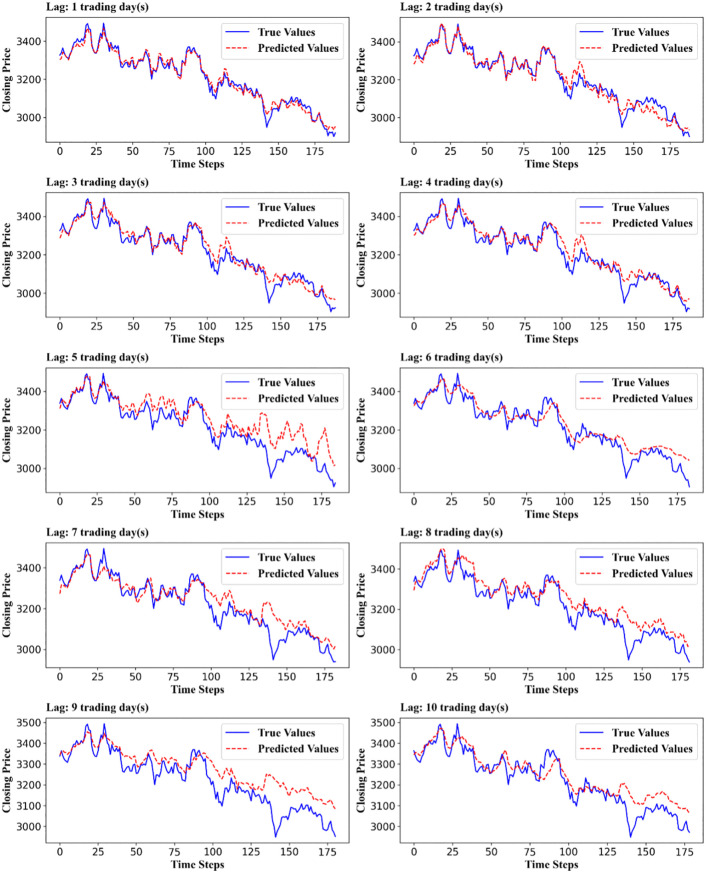
Forecast curves with a lag of 1-10 trading days. ***Note***: Figure 5 shows the predicted versus actual values of the SSE Composite Index across forecast horizons ranging from 1 to 10 trading days. The close alignment in the early lags indicates high short-term accuracy, whereas the gradual divergence at longer horizons reflects an increasing uncertainty over time.

Fig 5 shows the forecast curves of MSD-CNN2D-ABiLSTM for the SSE with lags of 1–10 trading days. Overall, the MSD-CNN2D-ABiLSTM demonstrates strong predictive ability in the short- to mid-term (lags of 1–10 days), particularly showing high accuracy and robustness in the first five days.

### 5.4. Structural sensitivity analysis

Structured Sensitivity Analysis (SSA) is a method for evaluating the impact of different model designs on performance. SSA facilitates the identification of architectural choices that improve the prediction accuracy, generalization ability, and computational speed for a given task by comparing different structural designs. The objective of SSA is to identify the key structural parts that affect model performance, thereby enabling more effective optimization. In our experiments, we conducted two structural tests to show that the important design decisions implemented in our model are both logical and effective in enhancing overall performance.

#### 5.4.1. Feature fusion test.

**Change**: Combine market measures (Mt$M_{t}$) and sentiment metrics (St$S_{t}$) into a single input set and transmit it directly to a CNN2D network.

**Purpose**: Determine whether separate handling of market and sentiment characteristics is required, and evaluate the usefulness of feature separation in improving model performance.

Tables 6 and 7 clearly show that the MSD-CNN2D-ABiLSTM model is better for short-term forecasting. For example, at a lag of 1, the model achieved a DA of 0.9153 and an R^2^ of 0. 0.9705, demonstrating its strong ability to capture short-term stock price trends. Although the feature fusion test performed reasonably well in the short term, its overall performance remained inferior to that of the MSD-CNN2D-ABiLSTM model.

**Table 7:** Performance statistics for the feature fusion test.

Lag	RMSE	MAE	R^2^	DA
1	0.0486	0.0399	0.9667	0.8783
2	0.0724	0.0530	0.9247	0.8989
3	0.0705	0.0582	0.9272	0.8449
4	0.1053	0.0785	0.8348	0.7957
5	0.1138	0.0787	0.8034	0.8152
6	0.1100	0.0815	0.8130	0.7978
7	0.1857	0.1475	0.4539	0.8187
8	0.1361	0.1168	0.7012	0.7845
9	0.0840	0.0654	0.8839	0.7667
10	0.1194	0.0963	0.7617	0.7360
11	0.1076	0.0859	0.8036	0.7345
12	0.1090	0.0865	0.7953	0.7443
13	0.1380	0.1104	0.6696	0.7486
14	0.1270	0.1001	0.7168	0.7241
15	0.2626	0.2357	−0.2293	0.6977
16	0.2034	0.1730	0.2497	0.7193
17	0.2112	0.1815	0.1778	0.6941
18	0.2213	0.1673	0.0819	0.6805
19	0.1781	0.1445	0.4008	0.6845
20	0.2215	0.1768	0.0688	0.6084

***Note****:* The length of the feature window was set to 10.

**Fig 6:**
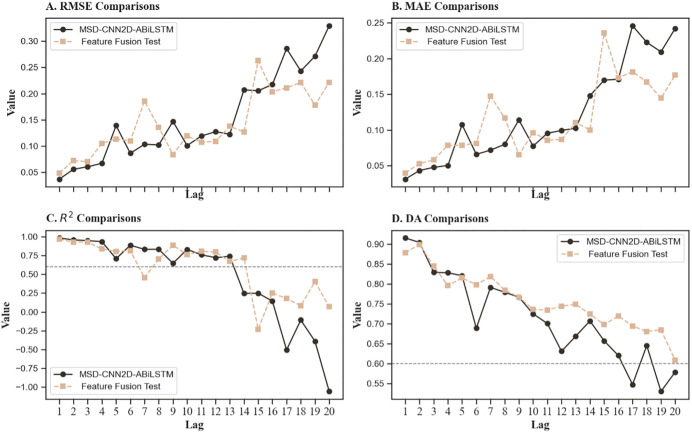
Comparative performance curves prior to and after feature fusion. ***Note*:** Compares the model’s predictive performance before and after feature fusion. The results show that separating market and sentiment features yields lower prediction errors (RMSE and MAE) and higher R^2^ values, indicating that distinct feature processing more effectively captures the heterogeneous mechanisms that drive market movements.

The performance comparison curves shown in Fig 6 further clarify the performance shifts before and after feature fusion. Clearly, within the effective prediction timeframe of 10 trading days, the MSD-CNN2D-ABiLSTM model consistently outperformed the post-fusion model in terms of the RMSE and MAE, indicating lower prediction errors. Additionally, the R^2^ values for the MSD-CNN2D-ABiLSTM model were higher, reflecting a better fit to the dataset. Although the post-fusion model slightly outperformed the MSD-CNN2D-ABiLSTM model in mid-term DA, the advantage of the MSD-CNN2D-ABiLSTM model was more pronounced in short-term forecasts, specifically at lags of 1 and 2.

In conclusion, the feature grouping strategy significantly enhances the model’s ability to accurately identify and predict stock price movements, as market technical and sentiment indicators impact prices through different mechanisms. Market technical features primarily reflect historical price fluctuations and technical factors, whereas sentiment features provide a unique perspective on the psychological expectations and emotional fluctuations of market participants. By combining these two sets of features, the MSD-CNN2D-ABiLSTM model demonstrates superior short-term trend forecasting capabilities.

#### 5.4.2. CNN type test.

**Change**: Without altering other aspects of the model structure, replace the CNN2D network with a CNN1D network.

**Purpose**: This modification will help determine whether local temporal features are more critical when handling complex multidimensional market data or whether the global dependencies between market and sentiment data play a more significant role in enhancing the model’s predictive performance.

**Fig 7:**
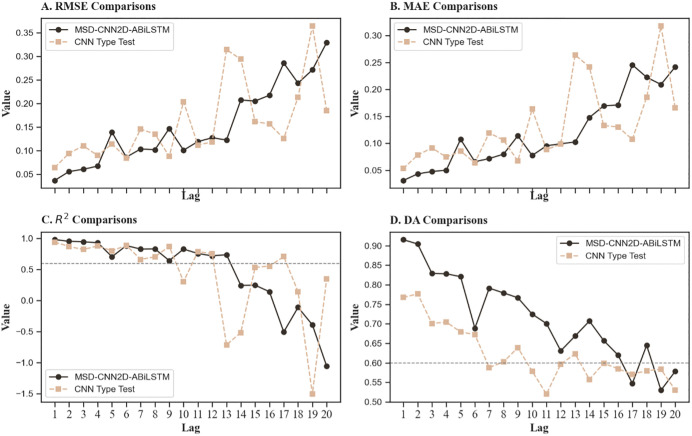
Comparative performance curves prior to and after CNN type change. ***Note***: Compares the model’s predictive performance when the CNN2D layer is replaced with a CNN1D layer. The results indicate that CNN2D achieves lower prediction errors and higher R^2^ values, confirming its superiority in capturing complex spatial dependencies within multidimensional feature spaces compared to CNN1D.

Table 8 presents the comparative results of the convolution layer type test. Fig 7, presenting data from Tables 6 and 8, shows the differences in model performance before and after the change. The research shows that the MSD-CNN2D-ABiLSTM model exhibits lower RMSE and MAE than the model that replaces CNN1D. This implies that the original model is significantly more effective in making predictions. A comparison of the R² values further proves the superior efficacy of the MSD-CNN2D-ABiLSTM model in explaining market data. Notably, the transition to CNN1D resulted in a significant decline in the model’s accuracy in predicting market trends. This suggests that when handling complex multidimensional data, CNN2D has a marked advantage over CNN1D owing to its superior ability to process features within a two-dimensional feature space.

**Table 8:** Performance statistics for the CNN type test.

Lag	RMSE	MAE	R^2^	DA
1	0.0645	0.0533	0.9413	0.7672
2	0.0947	0.0783	0.8712	0.7766
3	0.1103	0.0915	0.8217	0.7005
4	0.0898	0.0745	0.8798	0.7043
5	0.1145	0.0856	0.8010	0.6793
6	0.0848	0.0641	0.8889	0.6721
7	0.1458	0.1195	0.6632	0.5879
8	0.1354	0.1061	0.7044	0.6022
9	0.0882	0.0674	0.8720	0.6389
10	0.2038	0.1635	0.3056	0.5787
11	0.1122	0.0885	0.7863	0.5198
12	0.1186	0.0986	0.7576	0.5966
13	0.3139	0.2640	−0.7111	0.6229
14	0.2938	0.2419	−0.5148	0.5575
15	0.1615	0.1331	0.5349	0.5988
16	0.1567	0.1305	0.5547	0.5848
17	0.1259	0.1075	0.7077	0.5706
18	0.2137	0.1857	0.1438	0.5799
19	0.3642	0.3179	−1.5050	0.5833
20	0.1849	0.1660	0.3515	0.5301

*Note:* The length of the feature window was set to 10.

In conclusion, although CNN1D has strengths in processing one-dimensional time-series data, its ability to identify intricate inter-feature relationships is limited, which ultimately limits its short-term prediction accuracy compared to that of CNN2D. This highlights the importance of using CNN2D for more complex, multidimensional market data to enhance predictive performance.

#### 5.4.3. Model structure ablation experiments.

Model structure ablation experiments were conducted to evaluate the contributions of different temporal components to the overall performance. Three variants were designed: replacing the BiLSTM with a unidirectional LSTM, removing the attention mechanism, and introducing a Transformer-based encoder. All experiments used identical data and training settings to ensure comparability. This analysis helps reveal how each structural design—bidirectional recurrence, attention weighting, and transformer encoding—affects prediction accuracy and model stability.

A **LSTM vs. BiLSTM test**

In this test, the BiLSTM layers in the proposed MSD-CNN2D-ABiLSTM model were replaced with standard unidirectional LSTM layers to examine the role of bidirectional temporal modeling. The overall model structure, hyperparameters, and training procedures were maintained to ensure a fair comparison. All experiments were conducted under consistent settings, with a feature window length of 10 and forecast lags ranging from 1 to 20 trading days.

The results presented in Table 9 show that compared with the bidirectional model detailed in Table 6, the unidirectional LSTM model consistently yielded higher RMSE and MAE values, alongside lower R^2^ and DA metrics. These differences suggest that the bidirectional sequence learning mechanism plays a critical role in improving the model’s ability to capture temporal dependencies and enhance its predictive stability. By processing information from both past and future directions, the BiLSTM structure demonstrates a more comprehensive understanding of sequence dynamics, leading to stronger short-term forecasting performance.

**Table 9:** Performance statistics for the LSTM-based model.

Lag	RMSE	MAE	R^2^	DA
1	0.1296	0.1087	0.9408	0.7989
2	0.1879	0.1505	0.8733	0.7660
3	0.2095	0.1655	0.8393	0.5989
4	0.2380	0.1893	0.7889	0.6129
5	0.3083	0.2564	0.6396	0.4728
6	0.3139	0.2529	0.6190	0.6175
7	0.2349	0.1906	0.7814	0.4780
8	0.3037	0.2456	0.6280	0.4972
9	0.2797	0.2305	0.6783	0.4944
10	0.2488	0.1946	0.7412	0.5225
11	0.2848	0.2322	0.6557	0.4859
12	0.3533	0.2924	0.4625	0.4545
13	0.5108	0.4096	−0.1325	0.5029
14	0.5009	0.4239	−0.1008	0.5230
15	0.4245	0.3607	0.1967	0.4593
16	0.4167	0.3513	0.2131	0.4503
17	0.3457	0.2989	0.4490	0.4824
18	0.6226	0.4631	−0.8173	0.4793
19	0.5894	0.4643	−0.6403	0.4702
20	0.5152	0.4022	−0.2589	

*Note:* The length of the feature window was set to 10.

B **Model without the attention mechanism**

To assess the contribution of the attention mechanism to the overall predictive performance, the Attention layer in the MSD-CNN2D-ABiLSTM model was removed while maintaining all other structural and training settings unchanged. This modification enabled the model to rely solely on the sequential encoding from the BiLSTM layers without the adaptive feature-weighting process provided by attention. All experiments were conducted under the same configuration, with a feature window length of 10 and forecast lags ranging from 1 to 20 trading days.

As shown in Table 10, the model without the attention mechanism exhibits R² and DA values comparable to those of the baseline model detailed in Table 6, suggesting that the overall trend recognition remains largely similar. However, the removal of attention leads to a clear deterioration in the RMSE and MAE across nearly all forecast horizons. These results demonstrate that although attention does not significantly alter the model’s directional sensitivity, it plays a crucial role in reducing prediction errors and enhancing the overall stability of the MSD-CNN2D-ABiLSTM framework.

**Table 10:** Performance of the model without attention.

Lag	RMSE	MAE	R^2^	DA
1	0.0724	0.0584	0.9815	0.9153
2	0.1005	0.0846	0.9638	0.8357
3	0.1050	0.0849	0.9596	0.7807
4	0.1159	0.0898	0.9499	0.8295
5	0.2195	0.1633	0.8172	0.8370
6	0.2972	0.2638	0.6585	0.7924
7	0.1893	0.1465	0.8581	0.8022
8	0.2389	0.1780	0.7698	0.7624
9	0.4064	0.3076	0.3207	0.7778
10	0.2969	0.2304	0.6313	0.7079
11	0.7086	0.6171	−1.1316	0.6271
12	0.2201	0.1784	0.7914	0.6989
13	0.2165	0.1802	0.7966	0.5486
14	0.2382	0.1939	0.7510	0.6379
15	0.4736	0.3909	0.0001	0.6395
16	0.3961	0.3491	0.2890	0.6433
17	0.3523	0.3110	0.4278	0.6176
18	0.4858	0.4203	−0.1063	0.6509
19	0.4692	0.4081	−0.0398	0.6429
20	0.4403	0.3888	0.0806	0.6325

***Note****:* The length of the feature window was set to 10.

C **Model with transformer module**

To further evaluate the adaptability of the model structure and respond to recent advances in time-series modeling, the BiLSTM layers in the MSD-CNN2D-ABiLSTM model were replaced with a Transformer module.

However, because the proposed framework is built on a dual-anchoring feature structure—where market and sentiment matrices are separately encoded via CNN2D—the Transformer was not used as the core encoder. Instead, it was employed as a comparative temporal modeling component following the CNN2D feature extraction stage. This design enables a direct comparison between recurrent and self-attention-based temporal representations, thereby testing the robustness and generalization capability of the proposed model under consistent experimental settings (window size = 10; lags = 1–20).

As shown in Table 11, the Transformer-based variant performed substantially worse than the baseline MSD-CNN2D-ABiLSTM model detailed in Table 6. Across nearly all forecast horizons, the RMSE and MAE increased sharply, whereas R^2^ and DA fluctuated irregularly, even becoming negative at certain lags (e.g., lags 7 and 9). These results indicate that the Transformer module struggles to capture the short-term temporal dependencies embedded in the current data configuration.

**Table 11:** Performance of the model with a Transformer module.

Lag	RMSE	MAE	R^2^	DA
1	0.3035	0.2419	0.6750	0.6878
2	0.4812	0.4291	0.1694	0.6862
3	0.2408	0.1950	0.7877	0.4759
4	0.3167	0.2570	0.6262	0.4677
5	0.4127	0.3546	0.3539	0.6576
6	0.2783	0.1928	0.7006	0.6776
7	0.6717	0.5316	−0.7867	0.0000
8	0.1929	0.1558	0.8500	0.6022
9	0.6553	0.5196	−0.7662	0.0000
10	0.2291	0.1865	0.7805	0.3202
11	0.2479	0.2022	0.7391	0.4294
12	0.2383	0.1954	0.7555	0.5795
13	0.2118	0.1688	0.8052	0.6057
14	0.2837	0.2295	0.6470	0.5977
15	0.3746	0.3114	0.3746	0.6453
16	0.2550	0.2066	0.7052	0.4737
17	0.3092	0.2604	0.5593	0.7000
18	0.4390	0.3618	0.0964	0.6686
19	0.3493	0.2748	0.4239	0.5000
20	0.3814	0.2980	0.3102	0.5663

***Note****:* The length of the feature window was set to 10.

We believe that this performance degradation primarily resulted from an adaptation mismatch between the Transformer’s self-attention mechanism and the dual-anchoring CNN2D feature structure adopted in this study. The Transformer is inherently suited for modeling long-range dependencies, whereas our framework emphasizes localized, short-horizon market and sentiment interactions. Consequently, the global attention patterns of the Transformer may not align well with the locally encoded CNN2D features, leading to unstable temporal learning under limited data conditions.

Additionally, a portion of the degradation may be attributable to the feature encoding characteristics of the CNN2D module, which may have restricted the Transformer’s ability to fully exploit global dependencies. However, considering the main focus of this study, we did not conduct further verification on this aspect.

### 5.5. Sliding window experiment

We conducted all the aforementioned experiments using a feature window length of 10. To further evaluate the impact of the window length on the model’s predictive performance, we performed an adjustment experiment by exploring different window lengths, such as 20 and 30, and their effects on predicting closing prices with lags ranging from 1 to 20 trading days. This experiment aimed to examine the model’s dependence on the length of historical data to determine the optimal window configuration that enhances the model’s prediction accuracy and generalization ability.

**Fig 8:**
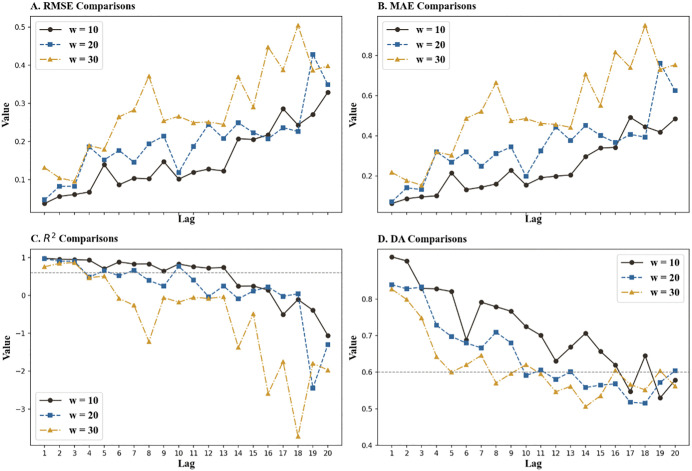
Comparative curves of model predictive performance across various feature windows. ***Note***: Compares the model’s predictive performance under different feature window lengths (w = 10, 20, 30). The results indicate that a shorter window (w = 10) achieves lower RMSE and MAE with a higher R² and directional accuracy (DA), whereas increasing the window size introduces excessive historical noise that reduces prediction accuracy and generalization ability.

Fig 8, derived from Table 12, visually compares the changes in predictive performance, illustrating the model’s performance in predicting future closing prices over the next 1–20 trading days, using different feature window lengths. According to the RMSE, MAE, R^2^, and DA measurements, setting the feature window length to 10 makes the model less prone to errors (RMSE and MAE) and more accurate (R^2^ and direction). However, as the window length increases, the model’s predictive performance gradually declines. This deterioration implies that longer windows may introduce excessive historical noise or irrelevant information, adversely affecting the model’s learning efficiency and weakening its generalization capability.

**Table 12:** Evaluation of model predictive efficacy across varying feature window sizes.

Lag	Window = 20				Window = 30			
	RMSE	MAE	R^2^	DA	RMSE	MAE	R^2^	DA
1	0.0417	0.0347	0.9756	0.8289	0.0904	0.0768	0.8851	0.8000
2	0.0942	0.0780	0.8729	0.8172	0.1311	0.1165	0.7542	0.8098
3	0.1096	0.0794	0.8245	0.7730	0.1922	0.1742	0.4607	0.7705
4	0.1063	0.0877	0.8321	0.7663	0.1141	0.0883	0.8066	0.6923
5	0.1761	0.1404	0.5300	0.7637	0.2271	0.2041	0.2209	0.5889
6	0.0899	0.0707	0.8751	0.6740	0.2324	0.2123	0.1680	0.6480
7	0.1352	0.1170	0.7108	0.6111	0.2193	0.1966	0.2416	0.5506
8	0.2203	0.2000	0.2177	0.6872	0.3359	0.3155	−0.8129	0.5763
9	0.1313	0.1150	0.7167	0.6573	0.2111	0.1824	0.2700	0.6136
10	0.1888	0.1598	0.4045	0.6420	0.4696	0.4281	−2.6662	0.5287
11	0.2432	0.1933	−0.0040	0.6171	0.4012	0.3788	−1.7168	0.5549
12	0.1928	0.1663	0.3604	0.5920	0.3384	0.3240	−0.9590	0.5640
13	0.3121	0.2870	−0.6901	0.5838	0.3175	0.2959	−0.7389	0.5906
14	0.2089	0.1853	0.2349	0.6221	0.3827	0.3602	−1.5525	0.5765
15	0.3493	0.2968	−1.1652	0.5882	0.4692	0.4509	−2.8802	0.6131
16	0.3264	0.2569	−0.9221	0.5799	0.4274	0.4110	−2.2728	0.5868
17	0.3661	0.3286	−1.4592	0.6429	0.3141	0.2922	−0.7976	0.5361
18	0.4260	0.3733	−2.3866	0.6287	0.4209	0.4063	−2.2824	0.6061
19	0.2744	0.2366	−0.4154	0.6084	0.3105	0.2806	−0.7989	0.5915
20	0.4796	0.4443	−3.3336	0.5915	0.3536	0.3294	−1.3410	0.5679

### 5.6. Sentiment feature ablation experiment

We also performed feature ablation experiments to determine the effect of sentiment features on SSE prediction. We evaluated the model’s predictive performance by removing sentiment group features (${IS}_{t}=\{is_{t},il_{t}\}$) and AG index features ($A_{t}=\{{ag}_{t},al_{t}\}$) from the input feature matrix. Here, ${IS}_{t}$ represents investors’ confidence in the market at time point t, whereas $A_{t}$ describes the level of investor divergence at the same time point. Together, these two components constitute the primary sentiment feature dimensions.

Table 13 presents the predictive performance of the model after feature ablation. By comparing the results in Table 13 with those in Table 6, we assessed the predictive performance of the model prior to and after the removal of the ${IS}_{t}$ features. The comparison shows that the original model outperforms the model that lacks the ${IS}_{t}$ feature group in terms of RMSE, MAE, $R^{\text{2}}$, and DA over the effective prediction timeframe of 10 trading days. When the ${IS}_{t}$ feature group is included, both the RMSE and MAE values are lower, indicating a smaller deviation between the predicted results and actual values, as well as a higher degree of data fitting.

**Table 13:** Predictive performance statistics after sentiment feature ablation.

Lag	Investor sentiment feature ablation				Agreement index feature ablation			
	RMSE	MAE	$R^{2}$	DA	RMSE	MAE	$R^{2}$	DA
1	0.0460	0.0348	0.9701	0.8148	0.0466	0.0353	0.9694	0.8783
2	0.0708	0.0572	0.9280	0.8564	0.0390	0.0305	0.9782	0.8457
3	0.0666	0.0535	0.9350	0.8003	0.0683	0.0532	0.9317	0.8503
4	0.0721	0.0529	0.9224	0.7887	0.0559	0.0455	0.9535	0.8441
5	0.1323	0.0980	0.7336	0.6772	0.0699	0.0536	0.9259	0.7663
6	0.1557	0.1216	0.6396	0.6096	0.1162	0.0946	0.7913	0.7650
7	0.0965	0.0729	0.8525	0.7747	0.1298	0.0938	0.7332	0.6813
8	0.1947	0.1801	0.3885	0.7624	0.1268	0.0906	0.7405	0.7348
9	0.1408	0.1162	0.6740	0.7111	0.1254	0.1036	0.7411	0.7278
10	0.1420	0.1246	0.6627	0.7921	0.1819	0.1574	0.4465	0.7978
11	0.1443	0.1166	0.6462	0.7345	0.1386	0.1123	0.6739	0.6441
12	0.1604	0.1358	0.5569	0.6875	0.1612	0.1404	0.5527	0.6477
13	0.1311	0.1116	0.7015	0.5714	0.1318	0.1101	0.6985	0.5714

*Note:* The length of the feature window was set to 10.

Furthermore, the model demonstrates a significantly better grasp of short-term market trends compared to the model without the ${IS}_{t}$ features. This analysis underscores the crucial role of the ${IS}_{t}$ features (which encapsulate relevant information about investor sentiment) in short-term stock market trend prediction, effectively enhancing the model’s predictive accuracy and trend-capturing ability.

**Fig 9:**
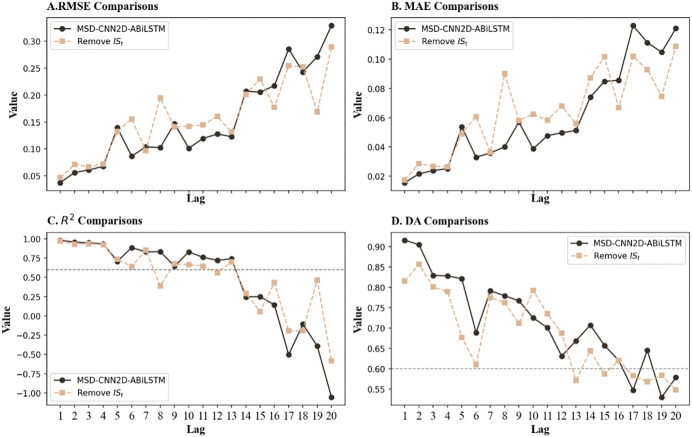
Performance comparison of ${𝐈𝐒}_{t}$ features before and after ablation. ***Note***: Illustrates the predictive performance before and after removing the investor sentiment feature set (*IS*_*t*_). The results show that excluding *IS*_*t*_ leads to higher prediction errors and lower R^2^ values, indicating that investor sentiment features substantially enhance short-term forecasting accuracy and improve the model’s ability to capture market trend dynamics.

Fig 9 visually compares the predictive performance before and after removing the investor sentiment feature set (${IS}_{t}$). The results show that excluding ${IS}_{t}$ leads to higher prediction errors and lower R^2^ values, indicating that investor sentiment features substantially enhance short-term forecasting accuracy and improve the model’s ability to capture market trend dynamics.

**Fig 10:**
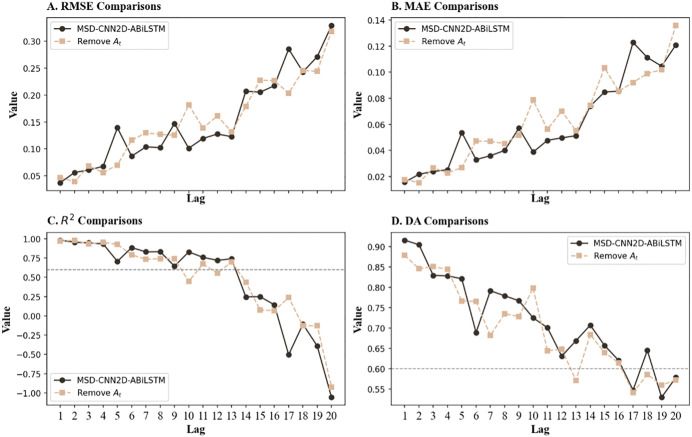
Performance comparison of ${𝐀}_{t}$ features before and after ablation. ***Note****:* Compares the model’s predictive performance before and after removing the agreement index feature set (${𝐀}_{t}$). The findings suggest that although ${𝐀}_{t}$ contributes little to short-term accuracy, it enhances medium-term forecasting performance by capturing the delayed effects of investor disagreement, reflecting its potential value in longer-horizon market trend prediction.

In addition to examining investor sentiment, we investigated the impact of investor disagreement (*A_t*) on model performance. Fig 10 presents the results of the $A_{t}$ ablation test, comparing predictive accuracy before and after removing this feature group. In short-term forecasts (lags of 1–5), deleting $A_{t}$ produces only a slight decrease in R^2^ and a minor increase in RMSE, suggesting that disagreement information contributes little to immediate market prediction. However, for medium-term forecasts (lag > 5), including $A_{t}$ helps reduce prediction error and enhances model fit, implying that investor disagreement exerts delayed effects on market dynamics.

Overall, although $A_{t}$ provides limited value in short-term forecasts owing to its non-directional nature, its inclusion improves the model’s robustness in medium-term trend estimation. This finding highlights the complementary role of sentiment agreement and disagreement features in capturing both immediate and gradual behavioral influences within the market.

## 6. Discussion

This section builds upon extensive experiments that validated the predictive effectiveness of the proposed model, offering a summary and discussion of the study’s key findings and implications. Specifically, this section is structured into three parts: (1) the construction logic and mechanism of the proposed model, (2) its theoretical and practical implications, and (3) its limitations and future directions.

### 6.1. Theoretical logic and structural mechanism

Building upon the extensive empirical results and validation experiments, this section further explains the theoretical rationale and structural logic underlying the proposed model. Rather than merely improving prediction accuracy, the study aimed to reveal the behavioral and structural mechanisms behind market fluctuations and to extend existing theories of sentiment-driven financial dynamics.

(1) **Theoretical motivation and research uniqueness**

The theoretical foundation of this study lies in advancing the understanding of emotional mechanisms within financial markets. Prior research has established that investor sentiment plays an essential role in price formation; however, most studies have examined either market anchoring or sentiment reactions separately, without integrating their interaction within a unified analytical framework. To address this gap, the present study introduces a dual-anchoring mechanism, in which market and sentiment signals jointly shape the evolution of stock prices.

This dual-anchoring concept extends traditional anchoring theory by linking rational market indicators with behavioral sentiment cues. It posits that market movements are neither purely rational adjustments nor spontaneous emotional reactions, but rather the outcome of continuous interaction between structural and affective forces.

Building upon this premise, the MSD-CNN2D-ABiLSTM model fuses market and sentiment anchors into one learning architecture, enabling simultaneous extraction of spatial and temporal dependencies. Unlike conventional CNN–BiLSTM hybrids that mainly rely on temporal correlations, our model explicitly captures cross-domain spatial coupling between price- and sentiment-based features. This represents an evolutionary extension of existing approaches rather than their replacement, refining prior hybrid frameworks to better reflect the complexity of sentiment-driven markets.

(2) **Construction and role of sentiment features**

Sentiment feature engineering is central to realizing the dual-anchoring logic. Building upon previous works [52,54], this study constructs two complementary indicators—the IS and AG indices—which jointly represent the intensity and convergence of market emotions. These indices form a bi-dimensional sentiment space, enabling the model to recognize both directional confidence and collective divergence among investors.

Importantly, neutral texts are excluded when computing sentiment indices. Although neutral sentiment may theoretically provide contextual balance, prior studies [50,51] have demonstrated that neutral expressions often contain semantic ambiguity and informational noise, contributing little to predictive performance. Following this empirical evidence, neutral posts are retained in preprocessing to preserve corpus integrity but omitted during index calculation. This ensures that sentiment variables capture only unambiguous bullish or bearish signals, thereby enhancing interpretability and reducing noise propagation through the learning pipeline.

The results confirm that incorporating sentiment anchors markedly improves both accuracy and directional prediction. The IS index reflects market confidence, whereas the AG index captures opinion divergence; together, they anchor collective behavior within broader market cycles. Hence, the dual sentiment structure not only strengthens short-term forecasting but also reveals the embedded psychological dynamics that govern long-term market equilibria.

(3) **Model innovation and structural rationality**

Methodologically, the MSD-CNN2D-ABiLSTM model innovatively integrates spatial feature extraction, bidirectional temporal learning, and adaptive attention weighting. The CNN2D module identifies localized spatial dependencies within market and sentiment matrices, the BiLSTM captures forward- and backward-time dynamics, and the Attention layer adaptively emphasizes the features most relevant to prediction. This layered synergy balances the market anchor (rational structure) with the sentiment anchor (behavioral dynamics), producing a model capable of learning both stable trends and emotional fluctuations.

Extensive experiments—which included comparisons with 19 baseline models, feature-ablation tests, and sliding-window sensitivity analyses—confirmed the model’s robustness and rational design. The model performs particularly well in the SSE, a market characterized by high retail participation and strong sentiment volatility. We interpret this not as a structural limitation but as an indication that the model aligns closely with markets where emotions significantly influence price discovery.

While emotional intensity differs across markets, sentiment mechanisms are universally present. Future research may extend validation to include markets with varying rationality levels. This would involve exploring cross-market adaptability and refining the understanding of how emotional heterogeneity shapes predictive efficiency.

Overall, the MSD-CNN2D-ABiLSTM framework embodies both theoretical and methodological advancements. By unifying rational and affective anchors, it offers a transparent and interpretable structure for modeling sentiment-driven dynamics—thereby extending anchoring theory into the domain of data-driven financial forecasting and enriching the analytical toolkit for understanding market behavior.

### 6.2. Research implications

#### 6.2.1. Theoretical significance.

This study innovatively introduced the concept of dual anchoring of market prices and sentiment into stock market prediction, expanding the application scope of anchoring theory. By integrating market price levels with investor sentiment characteristics, the study highlighted the importance of sentiment in market fluctuations, offering a new theoretical perspective for understanding the complex dynamics of financial markets.

Additionally, structural sensitivity tests, feature ablation experiments, and window sliding tests proved that the model performed well across different situations. These tests showed that CNN2D is particularly adept at handling complex spatial feature matrices. In addition, feature ablation experiments further emphasized the critical role of emotional features, although the predictive value of the AG index exhibited more complex features. These findings provide rich theoretical support and methodological reference for future research.

#### 6.2.2. Practical significance.

This study holds significant practical value for investors, regulators, and academic researchers. Investors can leverage the MSD-CNN2D-ABiLSTM model to make more accurate short-term investment decisions, thereby reducing risks associated with emotional volatility and enhancing the stability of returns. Moreover, regulators can use sentiment-driven predictive models to promptly identify abnormal market fluctuations, thereby optimizing regulatory policies and enhancing overall market stability. Researchers can build upon this study to explore other feature combinations and model optimizations, laying the groundwork for future stock market prediction research.

In conclusion, this study not only deepens the theoretical understanding of sentiment-driven market behavior but also provides effective solutions for real-world financial market operations. By using both market and sentiment as anchors, it demonstrate the significant impact of sentiment on market trends, providing strong support for investment decisions, risk management, and policymaking in severe financial market conditions.

### 6.3. Limitations

Although the proposed MSD-CNN2D-ABiLSTM model demonstrates robust performance in predicting short-term fluctuations of the SSE Composite Index, it has several limitations.

#### 6.3.1. Impact of external factors.

The study period (2019–2023) coincided with the COVID-19 pandemic, which profoundly affected both market dynamics and investor sentiment orientation in the Chinese stock market. This unprecedented event amplified emotional volatility, policy uncertainty, and behavioral divergence among investors, resulting in a market environment characterized by strong sentiment fluctuations and heightened noise. While such exogenous shocks may introduce instability and limit the model’s generalizability to more tranquil market conditions, they also provide a valuable opportunity to examine the model’s robustness under real-world stress and crisis scenarios.

Note that our data collection was completed in 2023, as the research project officially commenced in 2024, and 2023 represented the most recent complete and verifiable annual dataset available at that time. The inclusion of this dataset ensured that the model was trained and validated on the most up-to-date and behaviorally representative sentiment data, thereby improving the contemporaneity and practical relevance of the findings. Nevertheless, future studies could extend the temporal scope to include pre- and post-pandemic periods or explicitly incorporate external shock variables—such as macroeconomic uncertainty indices or crisis markers—to further evaluate the model’s adaptability and predictive stability across different market regimes.

**Limitations of sentiment features:** While the study explored the dual anchoring of market and sentiment, the sentiment features employed were relatively narrow in scope. The current model relies heavily on sentiment indices derived from social media text data, which may not comprehensively capture market sentiment dynamics. Future studies could expand the sentiment feature set by incorporating additional dimensions, such as investor sentiment surveys and broader market sentiment indices, to improve the comprehensiveness and accuracy of sentiment data and enhance the model’s ability to detect sentiment-driven market fluctuations.

**Trade-off between local and global analyses:** The study highlights the importance of local spatial features in predicting stock prices, particularly concerning short-term fluctuations. Nonetheless, the challenge of balancing the capture of local features with the analysis of broader market trends persists, particularly in the context of long-term market prediction. Future research could introduce multi-level feature analyses that integrate both local and global market dynamics, offering a more comprehensive understanding of market behavior and improving long-term forecasting capabilities.

#### 6.3.2. Geographic limitations.

Although the MSD-CNN2D-ABiLSTM model demonstrates robust performance in forecasting the Shanghai Composite Index, its results are significantly influenced by the behavioral characteristics of the Chinese stock market—such as high levels of retail participation, pronounced emotional sensitivity, and strong policy influence. These features make the SSE a suitable empirical setting for testing sentiment-driven forecasting frameworks. However, market rationality and emotional responsiveness vary across regions. In more mature markets, where institutional investors play a dominant role, the impact of sentiment may appear more moderate but is never entirely absent.

Future research should, therefore, conduct cross-market validation within equity, commodity, or foreign exchange markets to test the model’s robustness across diverse behavioral structures. Additionally, adaptively calibrating the influence of sentiment in accordance with market conditions could further enhance the model’s generalizability. Note that this limitation reflects differences in sentiment intensity rather than the scope of the model—as all markets, irrespective of their level of maturity, are influenced by emotion to some extent. Extending this framework to various market environments will contribute to developing a unified understanding of how sentiment interacts with market fundamentals in shaping financial dynamics.

In conclusion, we acknowledge these limitations as meaningful directions for future research. Further investigations that deepen the understanding of how sentiment and market fundamentals interact under different market structures will help extend the theoretical contribution of this study and promote a more systematic understanding of sentiment-driven financial dynamics.

## 7. Conclusion

This study examines the short-term prediction of the SSE Composite Index and introduces MSD-CNN2D-ABiLSTM, a multivariate feature matrix model that uses both market prices and investor sentiment as anchors. The model uses the highest and lowest closing prices and sentiment index extremes over a 52-week period as anchors, in conjunction with Fibonacci retracement levels, to create a composite feature set that characterizes market heterogeneity. The MSD-CNN2D-ABiLSTM model uses CNN2D to extract local spatial features from market and sentiment data, whereas a BiLSTM network is used to capture temporal features. The implementation of an attention mechanism enables the model to concentrate on critical information, thereby improving its capacity to forecast short-term variations in the SSE.

Experimental results show that the MSD-CNN2D-ABiLSTM model performs well in short-term SSE forecasting, particularly in predicting trends with a 1–2 day lag, with accuracy exceeding 90% and an R² value exceeding 95%. The model demonstrates a significant advantage in predicting stock market trends with a 10-day lag, markedly surpassing traditional baseline models. Feature ablation experiments validated the efficacy of the sentiment feature set, highlighting the significance of market sentiment in predicting the sentiment-driven Chinese stock market.

Additionally, structural sensitivity tests validated the enhancement in model performance by loading market and sentiment features independently. Furthermore, the study revealed that compared with CNN1D, CNN2D is more adept at handling complex market structures, with local spatial feature extraction proving particularly advantageous for short-term forecasting. The sliding window experiment highlights the critical role of the optimal window length in improving the model’s adaptability to various market conditions.

This research is the first to introduce dual anchoring of market prices and sentiment into SSE forecasting, enriching the theoretical framework of stock price prediction. It expands the application of anchoring effects in financial market forecasting while demonstrating the critical role of sentiment features in capturing market dynamics. By revealing the global dependencies between sentiment and market prices, this study provides a new perspective for the academic community and establishes a theoretical foundation for investigating anchoring effects and sentiment-driven market behavior.

The study also holds significant practical implications. Investors can utilize the MSD-CNN2D-ABiLSTM model to make more accurate short-term investment decisions, reducing the negative impact of emotional fluctuations on their decisions. Regulatory agencies can use the findings to identify abnormal sentiment-driven market volatility and optimize regulatory measures, thereby improving market stability. Researchers can build upon the methods and model design proposed in this study to further explore sentiment-driven market behavior analysis, advancing research in financial market prediction.

Despite its remarkable results in predicting the SSE, the study has several limitations. First, the model does not fully account for external factors such as macroeconomic conditions and policy interventions, which may limit its predictive power under extreme market conditions. Second, the sentiment features are relatively narrow in scope, and future research could introduce more dimensions of sentiment data, such as sentiment surveys and market indices. Finally, the model’s applicability is primarily based on the Chinese market. For cross-market validation, future studies could extend this approach to other financial markets, improving the model’s generalizability.

## Supporting information

S1 Data This file contains the English-version datasets of the SSE index and the sentiment index. (ZIP)

## References

[1] Chen Y, Fang R, Liang T, Sha Z, Li S, Yi Y. Stock Price Forecast Based on CNN-BiLSTM-ECA Model. Scientif Programm. 2021;2021:2446543. doi: 10/gt67sz

[2] Chen Y-C, Huang W-C. Constructing a stock-price forecast CNN model with gold and crude oil indicators. Appl Soft Comput. 2021;112:107760. doi: 10.1016/j.asoc.2021.107760

[3] Deng S, Xiao C, Zhu Y, Tian Y, Liu Z, Yang T. Dynamic forecasting of the Shanghai Stock Exchange index movement using multiple types of investor sentiment. Appl Soft Comput. 2022;125:109132. doi: 10.1016/j.asoc.2022.109132

[4] Liu B, Yu Z, Wang Q, Du P, Zhang X. Prediction of SSE Shanghai Enterprises index based on bidirectional LSTM model of air pollutants. Exp Syst Appl. 2022;204:117600. doi: 10.1016/j.eswa.2022.117600

[5] Nisar TM, Yeung M. Twitter as a tool for forecasting stock market movements: A short-window event study. J Financ Data Sci. 2018;4:101–19. doi: 10.1016/j.jfds.2017.11.002

[6] Lin Z. Modelling and forecasting the stock market volatility of SSE Composite Index using GARCH models. Fut Generat Comput Syst. 2018;79:960–72. doi: 10.1016/j.future.2017.08.033

[7] Jiang J, Wu L, Zhao H, Zhu H, Zhang W. Forecasting movements of stock time series based on hidden state guided deep learning approach. Inform Proc Manag. 2023;60(3):103328. doi: 10.1016/j.ipm.2023.103328

[8] Abraham ER, Mendes dos Reis JG, Vendrametto O, de Oliveira Costa Neto PL, Carlo Toloi R, de Souza AE, et al. Time Series Prediction with Artificial Neural Networks: An Analysis Using Brazilian Soybean Production. Agriculture. 2020;10(10):475. doi: 10.3390/agriculture10100475

[9] Roondiwala M, Patel H, Varma S. Predicting Stock Prices Using LSTM. Int J Sci Res. 2017;6(4):1754–6. doi: 10.21275/art20172755

[10] Kahneman D. Thinking, fast and slow. New York (NY): Farrar, Straus and Giroux; 2011. 499 p.

[11] Givi J, Galak J. The “future is now” bias: Anchoring and (insufficient) adjustment when predicting the future from the present. J Experimen Soc Psychol. 2019;84:103830. doi: 10.1016/j.jesp.2019.103830

[12] George TJ, Hwang C. The 52‐week high and momentum investing. J Financ. 2004;59:2145–76. doi: 10/dr7r93

[13] Baker M, Pan X, Wurgler J. The effect of reference point prices on mergers and acquisitions. J Financ Econ. 2012;106(1):49–71. doi: 10.1016/j.jfineco.2012.04.010

[14] Liang H, Yang C, Zhang R, Cai C. Bounded rationality, anchoring-and-adjustment sentiment, and asset pricing. North Am J Econ Financ. 2017;40:85–102. doi: 10.1016/j.najef.2017.02.001

[15] Xu H, Chai L, Luo Z, Li S. Stock movement prediction via gated recurrent unit network based on reinforcement learning with incorporated attention mechanisms. Neurocomputing. 2022;467:214–28. doi: 10.1016/j.neucom.2021.09.072

[16] Piotroski JD. Value Investing: The Use of Historical Financial Statement Information to Separate Winners from Losers. J Account Res. 2000;38:1. doi: 10.2307/2672906

[17] Fama EF, French KR. The Cross-Section of Expected Stock Returns. J Financ. 1992;47(2):427. doi: 10.2307/2329112

[18] Fama EF, French KR. A five-factor asset pricing model. J Financ Econ. 2015;116(1):1–22. doi: 10.1016/j.jfineco.2014.10.010

[19] De Almeida LAG. Technical indicators for rational investing in the technology companies: The evidence of FAANG stocks. J Pengur. 2020;59:75–87.

[20] Murphy KJ. Chapter 38 Executive compensation. Handbook of Labor Economics. Elsevier; 1999. p. 2485–563.

[21] Nti IK, Adekoya AF, Weyori BA. A systematic review of fundamental and technical analysis of stock market predictions. Artif Intell Rev. 2020;53:3007–57. doi: 10/gkfmzc

[22] McNeil AJ. Modeling Financial Time Series With S-PLUS. J Am Stat Assoc. 2004;99(466):564–5. doi: 10.1198/jasa.2004.s336

[23] Sahoo PK, Charlapally K. Stock price prediction using regression analysis. Int J Sci Eng Res. 2015;6:1655–9.

[24] Fenghua W, Jihong X, Zhifang H, Xu G. Stock Price Prediction based on SSA and SVM. Proced Comput Sci. 2014;31:625–31. doi: 10.1016/j.procs.2014.05.309

[25] Illa PK, Parvathala B, Sharma AK. Stock price prediction methodology using random forest algorithm and support vector machine. Mat Today Proc. 2022;56:1776–82. doi: 10.1016/j.matpr.2021.10.460

[26] Du X, Hayes DJ, Yu CL. Dynamics of Biofuel Stock Prices: A Bayesian Approach. Am J Agri Econ. 2010;93(2):418–25. doi: 10.1093/ajae/aaq157

[27] Liu H, Long Z. An improved deep learning model for predicting stock market price time series. Digit Signal Process. 2020;102:102741. doi: 10.1016/j.dsp.2020.102741

[28] Bao W, Yue J, Rao Y. A deep learning framework for financial time series using stacked autoencoders and long-short term memory. PLOS ONE. 2017;12:e0180944. doi: 10/gbnfcf 10.1371/journal.pone.0180944 PMC5510866 28708865

[29] Xiaoyan L, Raga RC. BiLSTM Model With Attention Mechanism for Sentiment Classification on Chinese Mixed Text Comments. IEEE Access. 2023;11:26199–210. doi: 10.1109/access.2023.3255990

[30] Istiake Sunny MdA, Maswood MMS, Alharbi AG. Deep Learning-Based Stock Price Prediction Using LSTM and Bi-Directional LSTM Model. 2020 2nd Novel Intelligent and Leading Emerging Sciences Conference (NILES). 2020. pp. 87–92.

[31] Abu-Mostafa YS, Atiya AF. Introduction to financial forecasting. Appl Intell. 1996;6:205–13. doi: 10/fvg4s5

[32] Abdullah M, Hadzikadicy M, Shaikhz S. SEDAT: Sentiment and Emotion Detection in Arabic Text Using CNN-LSTM Deep Learning. 2018 17th IEEE International Conference on Machine Learning and Applications (ICMLA). 2018. p. 835–40.

[33] Cen L, Hilary G, Wei KCJ. The Role of Anchoring Bias in the Equity Market: Evidence from Analysts’ Earnings Forecasts and Stock Returns. J Financ Quant Anal. 2012;48(1):47–76. doi: 10.1017/s0022109012000609

[34] Campbell SD, Sharpe SA. Anchoring Bias in Consensus Forecasts and Its Effect on Market Prices. J Financ Quant Anal. 2009;44(2):369–90. doi: 10.1017/s0022109009090127

[35] Ribeiro MT, Singh S, Guestrin C. Anchors: High-Precision Model-Agnostic Explanations. AAAI. 2018;32.

[36] Kumar A, Lee CMC. Retail Investor Sentiment and Return Comovements. J Financ. 2006;61(5):2451–86. doi: 10.1111/j.1540-6261.2006.01063.x

[37] Chung S-L, Hung C-H, Yeh C-Y. When does investor sentiment predict stock returns? J Empiric Financ. 2012;19(2):217–40. doi: 10.1016/j.jempfin.2012.01.002

[38] Stambaugh RF, Yu J, Yuan Y. The long of it: Odds that investor sentiment spuriously predicts anomaly returns. J Financ Econ. 2014;114(3):613–9. doi: 10.1016/j.jfineco.2014.07.008

[39] Al-Nasseri A, Menla Ali F, Tucker A. Investor sentiment and the dispersion of stock returns: Evidence based on the social network of investors. Int Rev Financ Analys. 2021;78:101910. doi: 10.1016/j.irfa.2021.101910

[40] Guo K, Sun Y, Qian X. Can investor sentiment be used to predict the stock price? Dynamic analysis based on China stock market. Phys A Stat Mech Appl. 2017;469:390–6. doi: 10.1016/j.physa.2016.11.114

[41] Jin Z, Guo K, Sun Y, Lai L, Liao Z. The industrial asymmetry of the stock price prediction with investor sentiment: Based on the comparison of predictive effects with SVR. J Forecast. 2020;39(7):1166–78. doi: 10.1002/for.2681

[42] Jing N, Wu Z, Wang H. A hybrid model integrating deep learning with investor sentiment analysis for stock price prediction. Exp Syst Appl. 2021;178:115019. doi: 10.1016/j.eswa.2021.115019

[43] Liu Q, Lee W-S, Huang M, Wu Q. Synergy between stock prices and investor sentiment in social media. Borsa Istanbul Rev. 2023;23(1):76–92. doi: 10.1016/j.bir.2022.09.006

[44] Baker M, Wurgler J. Investor Sentiment in the Stock Market. J Econ Perspect. 2007;21(2):129–51. doi: 10.1257/jep.21.2.129

[45] Jiang S, Jin X. Effects of investor sentiment on stock return volatility: A spatio-temporal dynamic panel model. Econ Modell. 2021;97:298–306. doi: 10.1016/j.econmod.2020.04.002

[46] Chen G, Kim KA, Nofsinger JR, Rui OM. Trading performance, disposition effect, overconfidence, representativeness bias, and experience of emerging market investors. Behav Dec Mak. 2007;20(4):425–51. doi: 10.1002/bdm.561

[47] Li Y, Li W. Firm-specific investor sentiment for the Chinese stock market. Econ Modell. 2021;97:231–46. doi: 10.1016/j.econmod.2021.01.006

[48] Lan Y, Huang Y, Yan C. Investor sentiment and stock price: Empirical evidence from Chinese SEOs. Econ Modell. 2021;94:703–14. doi: 10.1016/j.econmod.2020.02.012

[49] Sharpe WF. Capital Asset Prices: A Theory of Market Equilibrium under Conditions of Risk. J Financ. 1964;19(3):425. doi: 10.2307/2977928

[50] Liu Q, Huang C, Liu Y, Son H. Purely sentiment-driven stock index trend forecast: A probability model based on social media sentiment space. Knowl-Based Syst. 2025;325:113985. doi: 10.1016/j.knosys.2025.113985

[51] Liu Q, Son H. Data selection and collection for constructing investor sentiment from social media. Humanit Soc Sci Commun. 2024;11:1–13. doi: 10/gtz557

[52] Liu Q, Son H. Methods for aggregating investor sentiment from social media. Humanit Soc Sci Commun. 2024;11:1–22. doi: 10/gt7tp8

[53] Cunningham P, Cord M, Delany SJ. Supervised Learning. In: Cord M, Cunningham P, editors. Machine Learning Techniques for Multimedia: Case Studies on Organization and Retrieval. Berlin, Heidelberg: Springer; 2008. p. 21–49.

[54] Antweiler W, Frank MZ. Is All That Talk Just Noise? The Information Content of Internet Stock Message Boards. J Financ. 2004;59(3):1259–94. doi: 10.1111/j.1540-6261.2004.00662.x

[55] Xiong X, Luo C, Ye Z. Stock BBS and trades: The information content of stock BBS. J Syst Sci Math Sci. 2017;37:2359. doi: 10.12341/jssms13312

[56] Li W, Qi F, Tang M, Yu Z. Bidirectional LSTM with self-attention mechanism and multi-channel features for sentiment classification. Neurocomputing. 2020;387:63–77. doi: 10.1016/j.neucom.2020.01.006

[57] Liu Q, Huang M, Zhao L, Lee W-S. The dispositional effects of holidays on investor sentiment: Therapeutic and hygienic. J Innovat Knowl. 2023;8(2):100358. doi: 10.1016/j.jik.2023.100358

[58] Liu Q, Wang X, Du Y. The weekly cycle of investor sentiment and the holiday effect-- An empirical study of Chinese stock market based on natural language processing. Heliyon. 2022;8(12):e12646. doi: 10.1016/j.heliyon.2022.e12646 36619447 PMC9816973

[59] Liu Q, Son H, Lee W-S. The game of lies by stock investors in social media: a study based on city lockdowns in China. Financ Innov. 2024;10(1). doi: 10.1186/s40854-023-00587-y

[60] Bollinger J. Using bollinger bands. Stocks Commodit. 1992;10:47–51.

[61] Seshu V, Shanbhag H, Rao SR, Venkatesh D, Agarwal P, Arya A. Performance Analysis of Bollinger Bands and Long Short-Term Memory(LSTM) models based Strategies on NIFTY50 Companies. 2022 12th International Conference on Cloud Computing, Data Science & Engineering (Confluence). 2022. p. 184–190.

[62] Fiorenza A, Vincenzi G. From Fibonacci Sequence to the Golden Ratio. J Mathemat. 2013;2013:1–3. doi: 10.1155/2013/204674

[63] Gaucan V. How to use Fibonacci retracement to predict forex market. J Knowl Manag Econ Inform Technol. 2011;1:1.

[64] Azzam NA, Batulan RA. Fibonacci Trading Strategy. In: Ramadani V, Alserhan B, Dana L-P, Zeqiri J, Terzi H, Bayirli M, editors. Research on Islamic Business Concepts. Singapore: Springer Nature Singapore; 2023. p. 347–356.

[65] Ismailiyan M. How to use social networks’ user-produced content in news: A case study of the BBC. Commun Res. 2015;22:129–48.

[66] Bhattacharya A, Romani M, Stern N. Infrastructure for development: meeting the challenge. CCCEP, Grantham Research Institute on Climate Change and the Environment and G. 2012;24:1–26.

[67] Cen L, Lu H, Yang L. Investor Sentiment, Disagreement, and the Breadth–Return Relationship. Manag Sci. 2013;59(5):1076–91. doi: 10.1287/mnsc.1120.1633

